# Photostable triphenylmethyl-based diradicals with a degenerate singlet-triplet ground state and strong photoluminescence†

**DOI:** 10.1039/d5sc03673a

**Published:** 2025-07-07

**Authors:** Mona E. Arnold, Anika Lebzelter, Philipp Thielert, Rémi Blinder, Jonas Schmid, Julia Zolg, Emanuele Spatola, Fedor Jelezko, Max von Delius, Sabine Richert, Alexander J. C. Kuehne

**Affiliations:** a Institute of Macromolecular and Organic Chemistry, Ulm University Albert-Einstein-Allee 11 89081 Ulm Germany alexander.kuehne@uni-ulm.de; b Institute of Physical Chemistry, University of Freiburg Albertstraße 21 79104 Freiburg Germany sabine.richert@physchem.uni-freiburg.de; c Institute for Quantum Optics, Ulm University Albert Einstein Allee 11 89081 Ulm Germany; d Center for Integrated Quantum Science and Technology (IQST), Ulm University Albert-Einstein-Allee 11 89081 Ulm Germany; e Institute of Organic Chemistry, Ulm University Albert-Einstein-Allee 11 89081 Ulm Germany

## Abstract

We present a new class of luminescent diradicals based on tris(trichlorophenyl)methyl (TTM) cores symmetrically bridged by indolocarbazole donors. These diradicals exhibit pure diradical character *y*_0_ and unprecedented photoluminescence quantum yields *ϕ* of up to 18%, addressing key challenges in the development of stable, emissive organic diradicals. Light emitting diradicals represent a formidable challenge for synthetic chemists; for applications as molecular color centers in quantum sensing and as emitters in optoelectronics. Unlike conventional approaches that require the conversion of closed-shell precursors, we directly couple brominated TTM radicals *via* Buchwald–Hartwig coupling. The magnetic and optical properties of the resulting molecules are comprehensively characterized by electron paramagnetic resonance EPR, UV-vis absorption, and photoluminescence spectroscopy. This work unites the robust photophysics of discrete TTM radicals with the electronic versatility of donor-bridged multi-spin systems, offering a promising design strategy for functional open-shell emitters.

## Abstract 2

TTM-based diradicals bridged by indolocarbazoles show high diradical character and up to 18% quantum yield, offering a promising route to stable, luminescent open-shell emitters with isoenergetic singlet and triplet ground states.

## Graphical abstract

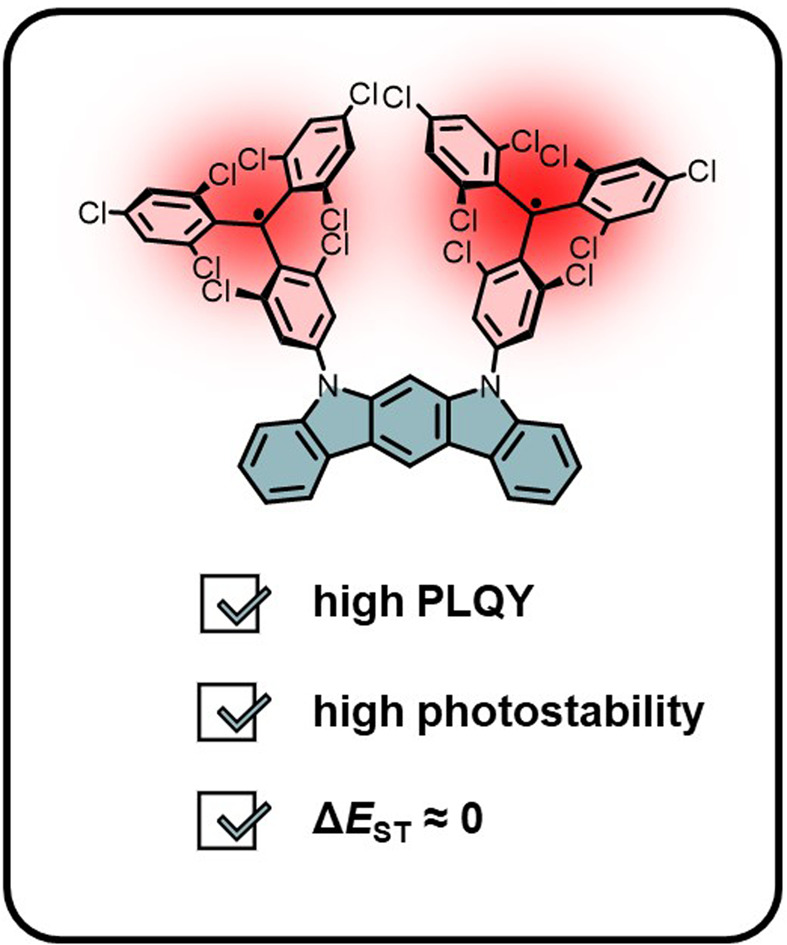

## Introduction

**Fig. 1:**
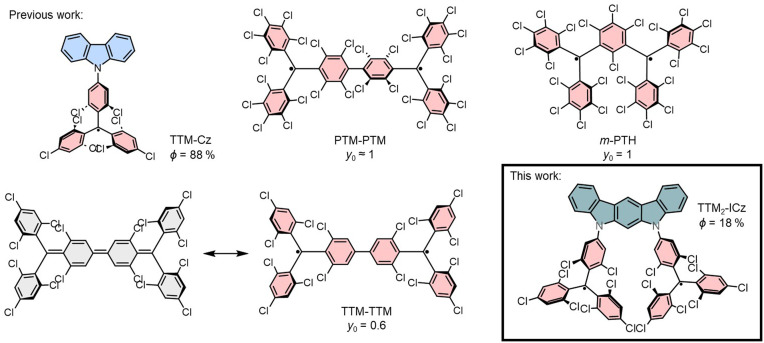
Previously established stable organic radicals, TTM-Cz with doublet GS and *ϕ* = 88%, Kekulé diradicals PTM–PTM and TTM–TTM with their respective *y*_0_ and singlet GS, and non-Kekulé *meta*-coupled *m*-PTH with triplet GS and 5,7-ICz-TTM_2_ with *ϕ* = 18% and degenerated singlet-triplet GS.

Quantum sensing offers unparalleled sensitivity compared to conventional sensors, enabling more precise detection of motion, as well as electric- and magnetic fields.^1–4^ This breakthrough promises transformative improvements in how we measure, navigate, observe, and interact with the world around us. Among quantum sensors, nitrogen-vacancy (NV) color centers in diamond stand out due to their luminescent spin-triplet manifold, which allows spin state initialization and readout using light.^5–7^ However, challenges such as precise positioning, scalability, and purity of NV color centers hinder their widespread application. By contrast, molecular color centers have been realized in metal complexes offering precise structural reproducibility and scalability through synthetic chemistry.^8^ Also organic molecules, such as triarylmethyl (trityl) radicals may be employed as viable spin carrying alternatives as they exhibit an unpaired radical electron. Chlorinated trityl radicals are exceptionally stable, owing to electronic effects and steric protection of the radical electron in their p-orbital.^9,10^ Functionalization with electron-donating groups – for example carbazole (Cz) – can render these monoradicals (with spin *S* = 1/2) highly fluorescent, achieving photoluminescence quantum yields *ϕ* as high as ≈ 90% (see Fig. 1, TTM-Cz).^11–14^ However, organic color centers can only be realized in molecules with spin *S* > 1/2. Trityl radicals can be linked to form diradicals, wherein two unpaired electrons in degenerate orbitals couple through dipolar and exchange interactions.^15–21^ These diradicals are categorized as Kekulé or non-Kekulé hydrocarbons. Formally, Kekulé diradicals exhibit an equilibrium between a closed-shell quinoidal and an open-shell diradical structure (with *y*_0_ representing the diradical character) with a singlet ground state (GS) and overall *S* = 0 (see Fig. 1, TTM–TTM, PTM–PTM).^15,20,21^ By contrast, non-Kekulé diradicals, such as Schlenk–Brauns radicals, exhibit a triplet ground state (*S* = 1), due to *meta*-positioning of the methine groups with their unpaired electrons *via* a central phenyl ring (see Fig. 1, *m*-PTH).^22,23^ Diradicals with a triplet ground state mimic the electronic structure of NV centers, making them promising candidates for quantum sensing and related technologies.^24^ Although trityl-based diradicals with accessible triplet states can generate ground-state polarization after optical excitation – an essential step toward enabling optically detected magnetic sensing – these molecules currently lack sufficient photoluminescence.^15,16,25,26^ This limitation restricts their application to ensemble measurements, precluding single-molecule readout, which is crucial for achieving the highest sensitivity and resolution. Developing fluorescent diradicals could bridge this gap, offering the reproducibility and scalability of synthetic chemistry alongside the functionality of molecular color centers.

Here, we report the synthesis and characterization of a new type of fluorescent diradical with high *ϕ* of 18%. The diradicals are derived from the tris(trichlorophenyl)methyl radical (TTM) motif but they differ from the typical Kekulé or Schlenk–Brauns geometry. Instead, we employ indolocarbazoles (ICz) as electron donating bridges, to which we attach two TTM radicals yielding stable diradicals with *y*_0_ ≈ 1. We study the influence of the relative orientation of the TTM radicals (with respect to the donor and each other) on the charge transfer (CT) character of the excited state (ES) and the *ϕ*. Moreover, the molecular arrangement of these diradicals influences the coupling between the radical electrons. The new donor-bridged diradicals introduced here represent a new approach and a successful realization of light emitting diradicals.

## Results and discussion

### Synthesis

**Fig. 2:**
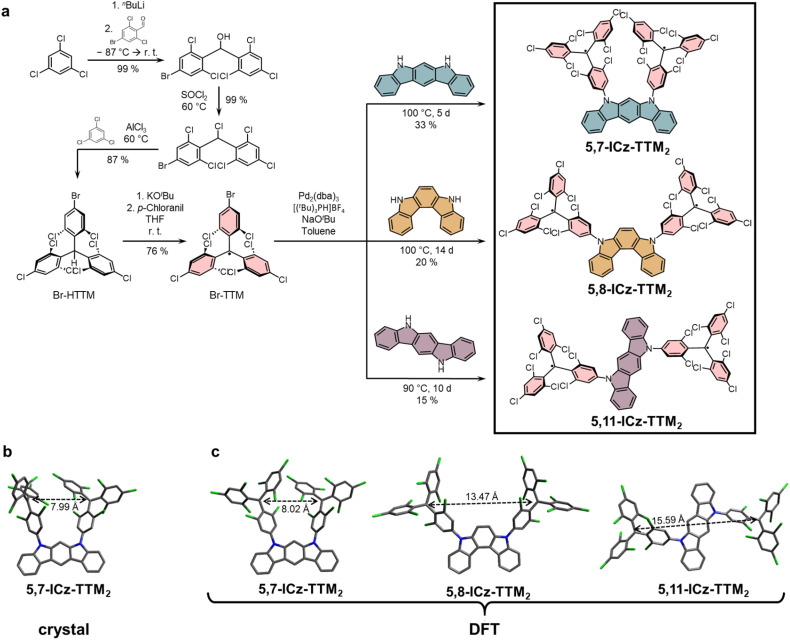
(a) Synthesis of indolocarbazole-bridged diradicals. Bromine-functionalized TTM is attached to the different indolocarbazoles by Buchwald–Hartwig coupling. The radical character is retained during the cross-coupling reaction. (b) Solid state structure of 5,7-ICz-TTM_2_, as determined by single-crystal XRay crystallography (diffusion of ^*n*^hexane into toluene, crystal system: triclinic, space group: *P*1̄) (c) DFT-calculated structures of 5,7-ICz-TTM_2_, 5,8-ICz-TTM_2_, and 5,11-ICz-TTM_2_ indicating the inter-spin distance and the relative orientation of the methine groups (level of theory: M06-L-GD3/def2-SVP with a CPCM solvation model to simulate a cyclohexane environment.^33–36^).

We synthesize three different indolocarbazole-bridged TTM diradicals by making use of the Buchwald–Hartwig cross-coupling reaction (see Fig. 2). Because the individual donor strength has crucial impact on how easily the methine group of the trityl moiety can be deprotonated and oxidized, the conversion of the closed-shell precursor is often challenging in donor-functionalized trityl radicals.^2,27,28^ Therefore, we first convert the triarylmethane precursor to the radical and couple the radicals directly to the indolocarbazole donors. The radicals remain intact during the Pd-catalyzed Buchwald–Hartwig cross-coupling reaction (*vide infra* EPR discussion).^29^ To improve the selectivity, we employ a novel *para*-bromine-functionalized TTM (Br-TTM) derivative (see Fig. 2). The closed-shell Br-HTTM precursor is obtained after a three-step synthesis in an overall high yield.^30,31^ The Br-TTM radical is obtained by following the most widely established protocol for TTM radical conversion, *via* deprotonation using KO^*t*^Bu, followed by mild oxidation using *p*-chloranil. The three diradicals are obtained after Buchwald–Hartwig coupling reactions of the Br-TTM to the different indolocarbazoles. While the yields are moderate, the diradicals are obtained pure and without the otherwise required deprotonation and oxidation steps in several repetitions.^15,26,32^

The diradicals are characterized by electron paramagnetic resonance (EPR) spectroscopy. Since the radical electrons typically deshield the nuclear spins in the molecules, we cannot perform NMR-spectroscopy on the open-shell molecules. That is why we also synthesize the closed-shell compounds, on which we perform ^1^H-NMR spectroscopy (see Experimental section and Fig. S39–S51 in the ESI†). The successful synthesis of the six molecules is also confirmed by mass spectrometry (see Fig. S33–S38 in the ESI†).

### Molecular geometry and inter-spin distance

Diradical 5,7-ICz-TTM_2_ has been successfully crystallized by solvent diffusion of ^*n*^hexane into a solution of 5,7-ICz-TTM_2_ in toluene. Single crystal XRay diffraction of 5,7-ICz-TTM_2_ allows its categorization into the centrosymmetric *P*1̄ space group (see Fig. 2). The central triphenylmethyl-carbon atom exhibits sp^2^ hybridization with a bond length of 1.43 to 1.44 Å to the three connected di- or trichlorophenyl rings, substantiating the pure diradical character (*y*_0_ ≈ 1) of 5,7-ICz-TTM_2_ (see Table 1). Moreover, from the crystal structure, an inter-spin distance of *d* = 7.99 Å is obtained. Interestingly, the two p-orbitals of the TTM radicals are oriented in an almost perpendicular fashion, which will minimize magnetic dipole coupling between the two radical electrons in the crystal.

**Table 1:** Photophysical and magnetic properties of the diradicals. Absorption (*λ*_abs_) and emission (*λ*_em_) maxima, molar absorption coefficients for the GS → ES transition (*ε* (GS-ES)), photoluminescence quantum yields *ϕ*, and photoluminescence lifetimes *τ* were measured in cyclohexane solutions (10^−4^ M). Values of the axial dipolar coupling, *dip*_ax_, were obtained from simulations of the EPR measurements performed in frozen toluene solutions at 80 K. The inter-spin distance *d* as well as *y*_0_ (for the (*P*, *M*) diastereomer) were obtained from DFT calculations. The inter-spin distance *d* has also been estimated from EPR data for comparison

Compound	*λ*_abs_/nm	*ε* (GS-ES)/10^3^ M^−1^ cm^−1^	*λ*_em_/nm	*ϕ*/%	*τ*/ns	*k*_r_/10^6^ s^−1^	*k*_nr_/10^6^ s^−1^	*y*_0_ (*P*, *M*)^a^	|Δ*E*_ST_|/kJ mol^−1^	dip_ax_/MHz	*d*/Å (DFT)	*d*/Å (EPR)
5,7-ICz-TTM_2_	641	6.00	680	18	15	12	55	0.98	0.052	83.4	8.02	8.5
5,8-ICz-TTM_2_	661	8.49	716	2	3	8	357	0.99	—	22.3	13.47	13.3
5,11-ICz-TTM_2_	674	4.13	720	2	3	8	357	0.97	—	17.5	15.59	14.4

Unfortunately, we were unable to crystallize the other two diradicals 5,8-ICz-TTM_2_ and 5,11-ICz-TTM_2_, which is why we perform DFT calculations for the three diradicals to investigate their structure and inter-spin distances. We optimize the molecules in their GS geometry at the M06-L-GD3/def2-SVP level of theory with a CPCM solvation model to simulate a cyclohexane environment.^33–36^ Single-point calculations are performed on the optimized structures employing PBE0-GD3/def2-TZVP.^34–37^ All three molecules exhibit a diradical character of *y*_0_ ≈ 1, indicating that the torsion around the TTM–N bond, effectively breaks conjugation and therefore making it irrelevant whether the nitrogens in the different ICz isomers are oriented towards each other in *para*- or *meta*-position (*cf.*Table 1 and Fig. 2). This is in stark contrast to Kekulé or Schlenk–Brauns diradicals, where the respective connectivity determines the singlet or triplet character of the GS. The calculated inter-spin distances increase from *d* = 8.02 Å for 5,7-ICz-TTM_2_ to 13.47 Å for 5,8-ICz-TTM_2_ and 15.59 Å for diradical 5,11-ICz-TTM_2_ (see Table 1). The calculated *d* of 5,7-ICz-TTM_2_ is in excellent agreement with the inter-spin distance observed experimentally in the crystal, whereas the perpendicular orientation of the radical p-orbitals is not reproduced in the DFT calculations, indicating that in solution the trityl units may have some degree of rotational freedom with respect to the ICz donor unit (see Fig. 2).

Within the crystal, the TTM propellers adapt a *P*,*M*-configuration with opposite helical chirality of the two TTM groups. We calculate the *P*,*P*- and *P*,*M*-configurations for 5,7-ICz-TTM_2_ as the diastereomers may have different physical properties. We do not calculate the molecule in the *M*,*M*- and *M*,*P*-configurations as they will behave analogously to their respective enantiomers. For small distances between the TTM moieties as in 5,7-ICz-TTM_2_, their relative orientation can induce strain and affect the total energy of the molecular system. Indeed, the *P*,*M*-configuration is stabilized by 7.6 kJ mol^−1^ with respect to the *P*,*P*-diastereomer. However, this small energy difference renders both isomers accessible in solution at room temperature.

### Electron paramagnetic resonance spectroscopy

**Fig. 3:**
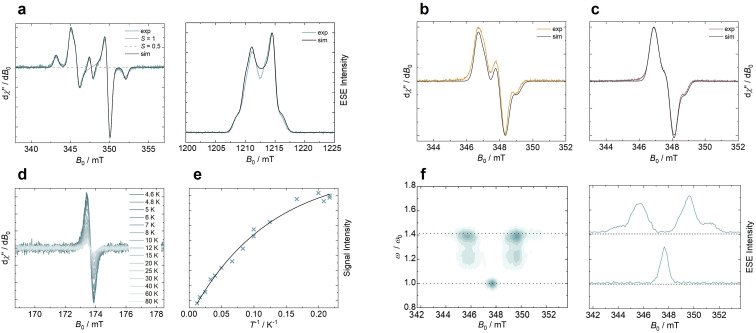
(a) Experimental cw-EPR X-band spectrum (left) and electron spin echo (ESE) detected Q-band EPR spectrum (right) of 5,7-ICz-TTM_2_ (turquoise) in frozen toluene solution (*T* = 80 K) with simulated spectra (black) using a triplet spin species. The central signal (dashed gray line) observed for 5,7-ICz-TTM_2_ corresponds to contamination with monoradical. Experimental cw-EPR X-band spectra of (b), 5,8-ICz-TTM_2_ (orange), and (c) 5,11-ICz-TTM_2_ (purple) measured for frozen toluene solutions of the diradicals at *T* = 80 K and simulated spectra (black) using a coupled doublet–doublet spin species. (d) Half-field transition (Δ*m*_*s*_ = ±2) spectra of 5,7-ICz-TTM_2_ in frozen toluene for different temperatures (left) and temperature-dependent signal intensity of the half-field transition, obtained from numerical double integration, and (e) Bleaney–Bowers fit of the VT-EPR data. (f) Fourier transform of the field-swept PEANUT data of 5,7-ICz-TTM_2_ referenced to the monoradical impurity signal at 347.7 mT (left) and the corresponding ESE spectra for *ω*_0_ and 
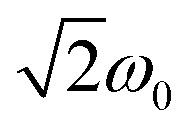
 (right), with the nutation frequency *ω* and the reference.

To characterize their magnetic properties, we perform electron paramagnetic resonance (EPR) spectroscopy of the three diradicals at the X- and Q-band in frozen toluene solution at *T* = 80 K (see Fig. 3a–c). Details on the setup and experimental parameters are given in the ESI.† A field-swept phase-inverted echo-amplitude detected nutation (PEANUT) experiment is carried out for 5,7-ICz-TTM_2_ (see Fig. 3f).^38^ The Fourier transform yields the nutation frequency of the spin species with the applied microwave pulse. A nutation frequency of 
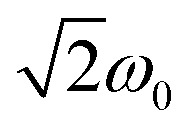
 is expected for a triplet spin species, with *ω*_0_ being a pure spin 1/2 (see Fig. 3f). The observed minor *S* = 1/2 contribution in 5,7-ICz-TTM_2_ at 347.7 mT is attributed to residual monoradical impurity (*ca.* 2.5% according to the simulated EPR spectrum), which we use as a reference (*vide infra* cw-EPR discussion). By this evaluation, the main signal of 5,7-ICz-TTM_2_ can be clearly attributed to a triplet spin species. The obtained spectra of 5,7-ICz-TTM_2_ are thus simulated as a spin triplet (*S* = 1), yielding an anisotropic *g*-tensor (*g* = 2.0026, 2.0042, 2.0037) and zero-field splitting parameters ǀ*D*ǀ = 125.2 MHz and ǀ*E*ǀ = 5.5 MHz.

To estimate the coupling strength for the three diradical compounds, the experimental spectra are simulated using a coupled spin system of two spin *S* = 1/2 (see ESI Fig. S29 and Table S3†). The axial dipolar coupling dip_ax_ increases from 5,11-ICz-TTM_2_ over 5,8-ICz-TTM_2_ to 5,7-ICz-TTM_2_, due to the decreased inter-spin distance (see Table 1). The dipolar coupling analysis can be used to obtain an experimental estimate of the inter-spin distance in the diradicals (see ESI† for details). These inter-spin distances closely match the distances between the radical centers determined using DFT, supporting the validity of the results and the suitability of our quantum chemical methods (see Table 1).

For 5,7-ICz-TTM_2_, we observe the formally forbidden half-field transition of the triplet spin state, confirming our assignment and the accessibility of the triplet state (see Fig. 3d). We further conduct variable-temperature (VT) EPR experiments between 4.6 and 80 K and plot the doubly-integrated signal intensities of the half-field transition against the temperature (see Fig. 3d and e). The Bleaney–Bowers fit reveals anti-ferromagnetic coupling indicating a slight stabilization of the singlet state *versus* the triplet state (see Section 6.3 in the ESI† for details).^39–43^ However, the energetic separation of singlet and triplet states |Δ*E*_ST_| is as low as 0.052 kJ mol^−1^ (0.012 kcal mol^−1^), indicating near-degeneracy of the two states. Due to the increased distance, the inter-spin coupling is reduced for 5,8-ICz-TTM_2_ and 5,11-ICz-TTM_2_, and |Δ*E*_ST_| is expected to be even smaller (see Table 1). For 5,8-ICz-TTM_2_, the intensity of the half-field transition is found to be reduced considerably as compared to 5,7-ICz-TTM_2_, while no half-field transition is observed for 5,11-ICz-TTM_2_, indicating that the two unpaired spins behave as isolated doublets rather than a coupled triplet state. These findings correlate well with the spin density maps, where we see that the unpaired electrons are mainly situated at the central methine carbon and slightly delocalized across the attached phenyl rings (see Fig. S27 in the ESI†).

### Optical characterization

**Fig. 4:**
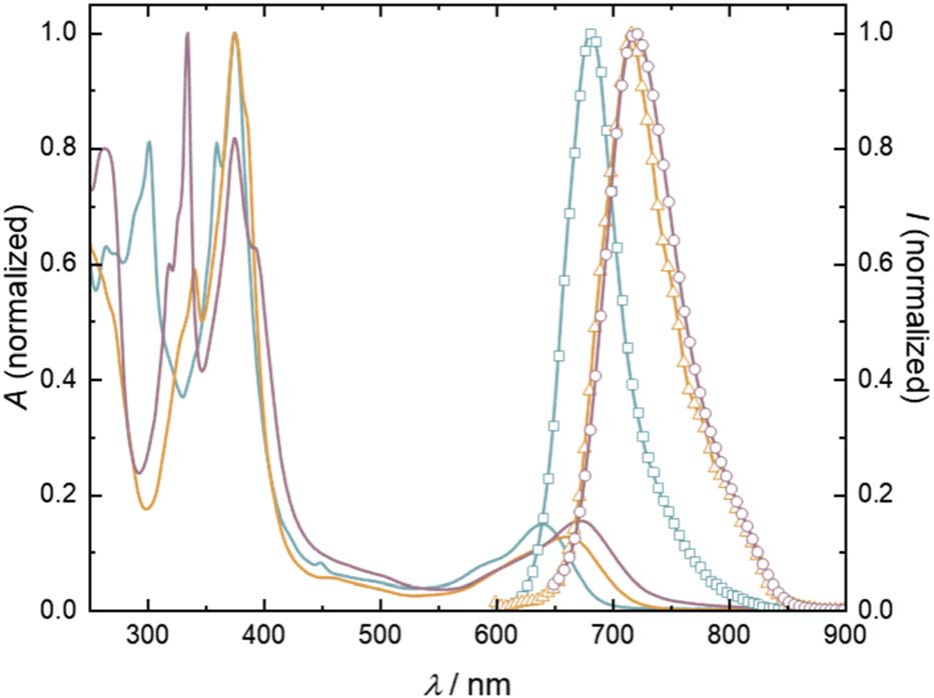
Absorption (solid lines) and emission (solid lines with symbols) spectra of the novel diradicals (5,7-ICz-TTM_2_ (turquoise, □), 5,8-ICz-TTM_2_ (orange, Δ), 5,11-ICz-TTM_2_ (purple, ○)) measured in cyclohexane solutions (10^−4^ M).

The new diradicals are also investigated using UV-vis absorption and photoluminescence spectroscopy. We employ cyclohexane as a non-polar solvent to prevent strong solvent–solute interactions. We observe absorption features in the UV region that can be related to the indolocarbazole donors in addition to an intense absorption peak in the UV and a rather weak absorption band in the visible region, as is typical for TTM-derived radicals (see Fig. 4).^11,14,44,45^

TD-DFT calculations on the previously optimized structures employing PBE0-GD3/def2-TZVP with a SMD solvent model yield calculated absorption spectra that are in excellent agreement with the experiment (see Fig. S24–S26 and Table S3 in the ESI†).^46^ We observe similar excitation energies for both triplet and broken-symmetry singlet states (see Fig. S24 & S26 in the ESI†). The *P*,*M*-configuration of 5,7-ICz-TTM_2_ is closer to the experiment than the *P*,*P*-configuration, which is in line with the slightly smaller energy of the former, favoring the formation of the mixed chirality diastereomer. Presumably, the experimental spectrum results from a mixture of both diastereomers. When we dissolve the crystals of diastereomerically pure 5,7-ICz-TTM_2_ in cyclohexane, we observed the same optical behavior as prior to crystallization, indicating that the TTM propellers can invert quickly at room temperature in solution (see Fig. S11†).

**Fig. 5:**
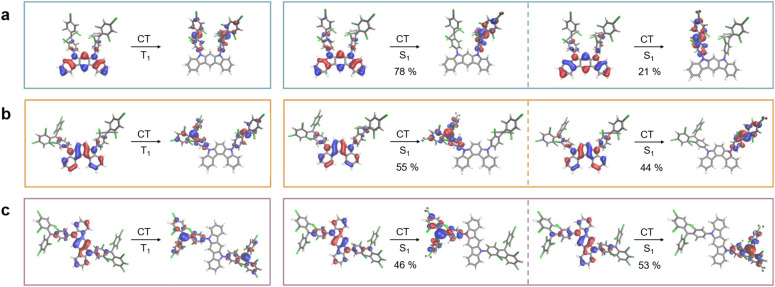
Natural transition orbitals (NTOs) for the GS→ES transition in (a) 5,7-ICz-TTM_2_, (b) 5,8-ICz-TTM_2_, and (c) 5,11-ICz-TTM_2_ in their GS geometry for triplet T_1_ (left) and broken-symmetry singlet S_1_ (right).

The natural transition orbitals (NTOs) confirm the nature of the electron donating ICz, yielding a clear CT excited state with the hole residing on the ICz moiety, whereas the electron is residing on the trityl units for both (singlet and triplet) spin states (see Fig. 5).

For the triplet state, electron density migrates from the ICz site to both singly occupied molecular orbitals (SOMOs) located on the TTM units (see Fig. 5). For the singlet state, the lowest ES is reflected by a linear combination of configurations resulting from charge migration from the highest doubly occupied molecular orbital (HDMO) to a single SOMO. Thus, the CT character of this mixed configurational exciton for the singlet is the same as observed for the triplet state (see Fig. 5). This behavior is similar for all three diradicals and in agreement with experimental and theoretical observations for the ES in related monoradicals.^11,14,47^ In other words, in the GS, the HDMO is located on the ICz, while the SOMOs reside on the TTM units. Thus, the excitation energy is determined by the electron donating capability of the ICz donor. We compare the energies of the highest occupied molecular orbital (HOMO) of the isolated ICz groups as a measure for their donor strength. When we calculate the isolated donors, we find a HOMO energy, which is 0.1 eV lower for 5,7-ICz than for 5,8-ICz and 5,11-ICz (see ESI†). Thus, their higher HOMO energy renders 5,8- and 5,11-ICz stronger electron donors than 5,7-ICz. As such, the ICz bridged TTM diradicals clearly behave similar to the TTM-Cz monoradicals, with their strong and solvent-dependent photoluminescence.^11,48^ Interestingly, the CT related bands in the visible spectrum appear at lower energy for 5,8-ICz-TTM_2_ and 5,11-ICz-TTM_2_, compared to 5,7-ICz-TTM_2_ (see Fig. 4 and Table 1). These characteristics correlate well with the above-described TD-DFT results and calculated HOMO energies and absorption spectra. The larger energy gap of diradical 5,7-ICz-TTM_2_ is also reflected in the photoluminescence spectra, where 5,7-ICz-TTM_2_ exhibits a maximum at 680 nm, whereas 5,8-ICz-TTM_2_ and 5,11-ICz-TTM_2_ show maxima in the NIR spectrum at 716 and 720 nm, respectively (see Fig. 4 and Table 1).

We determine *ϕ* for all three diradicals in cyclohexane solutions. Because of the high donor strength of 5,8-ICz and 5,11-ICz the quantum yield of the respective diradicals is *ϕ* = 2%, similar to monoradicals functionalized with donors of similar strength (see Table 1).^14^ By contrast, the emission of 5,7-ICz-TTM_2_ is considerably enhanced with a quantum yield of *ϕ* = 18%. We attribute this increased *ϕ* to the clear CT state upon excitation evoked by the 5,7-ICz of lower and therefore more appropriate donor strength than 5,8-ICz and 5,11-ICz in 5,8-ICz-TTM_2_ and 5,11-ICz-TTM_2_. When changing the solvent from cyclohexane to the slightly more polar toluene, all the three diradicals are rendered dark, further substantiating our conclusion that the ES exhibits a pronounced CT character.

### Excited state dynamics and photostability

The excited state kinetics of the diradicals are investigated to rationalize their different emission quantum yields. We employ photoluminescence lifetime studies to determine the rate constants for radiative (*k*_r_) and non-radiative (*k*_nr_) relaxation (see Table 1), using the following well-known relations:
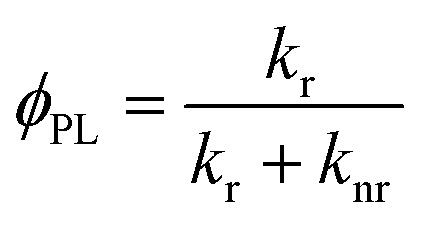

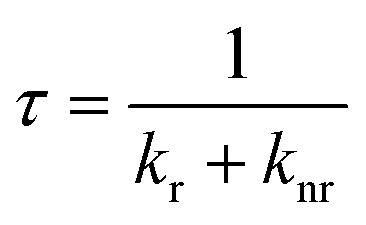


We find *k*_r_'s of the same order of magnitude for all three compounds, while the radiative rate constant is slightly higher for 5,7-ICz-TTM_2_. By contrast, *k*_nr_ is almost one order of magnitude smaller for 5,7-ICz-TTM_2_ compared to 5,8-ICz-TTM_2_ and 5,11-ICz-TTM_2_. Thus, the suppression of non-radiative relaxation is identified as the main reason for the improved *ϕ*. For related monoradicals, we have demonstrated that low-lying CT excited states can be deactivated efficiently through conical intersections with the potential energy surface of the GS.^13^ Therefore, *ϕ* drops in a series of carbazole-functionalized radicals with increased CT character of the ES. Simultaneously, the energy of the ES and thus the emission correlates with *ϕ*. Low-energy emission, as observed for 5,8-ICz-TTM_2_ and 5,11-ICz-TTM_2_ is typically connected to poor emission efficiency.^13^ Thus, we conclude that the difference in the ES energy between 5,7-ICz-TTM_2_, as compared to 5,8-ICz-TTM_2_ and 5,11-ICz-TTM_2_, results in its superior value of *ϕ*.

To investigate the photostability of the diradicals, we irradiate cyclohexane solutions with UV-light (*λ* = 395 nm, see Section 4 in the ESI† for details) and use TTM and TTM-Cz as references. Since the luminescent monoradical is a possible degradation product of our diradicals, we refrain from determining the photostability by photoluminescence and we use UV-vis spectroscopy instead (see Section 4 in the ESI† for details). Both reference compounds degrade completely within 3 seconds and 1 minute, respectively. By contrast, the diradicals have superior photostability. For 5,7-ICz-TTM_2_ we extract a half-life *t*_1/2_ = 19 min (5,8-ICz-TTM_2_: *t*_1/2_ = 34 min and 5,11-ICz-TTM_2_: *t*_1/2_ > 60 min), which indicates that the photostability is increased by more than two (to three) orders of magnitude compared to TTM-Cz and TTM monoradicals, respectively (see Section 4.4 of the ESI†). This highly improved photostability is likely related to the pronounced CT character of the ES. Superior stability has previously been reported for TTM-Cz derived radicals with red-shifted absorption and emission.^44^

## Conclusions

We have introduced a novel class of TTM-based diradicals employing indolocarbazole as a linker. In the series of investigated compounds, the inter-spin distance is systematically increased from 8 to 16 Å by choosing three different constitutional isomers bridging the trityl moieties. We observe a weak dipolar interaction between the unpaired electron spins leading to a degenerate singlet-triplet GS. For 5,7-ICz-TTM_2_ with its short radical–radical distance and clear CT excited state, the diradical exhibits an emission peaking at 680 nm with *ϕ* = 18%, which is one of the highest reported values for TTM-derived diradicals (see Table S2 in ESI†). When the TTM groups are attached to 5,8-ICz and 5,11-ICz of higher donor strength, the emission is shifted to the near-infrared region. We find the 5,7-ICz derivative to be at least 2–3 orders of magnitude more photostable, than related monoradicals. These findings on emission wavelength, quantum yield, and photostability are essential for the design of future fluorescent diradicals. While the spin–spin interaction in the investigated diradicals is too weak to lift the degeneracy of the GS, the *N*-donor-bridged diradicals serve as model systems to understand the emission properties of future related compounds. A triplet ground state might be achieved by further decreasing the inter-spin distance while retaining the optical properties, whereas higher *ϕ* may be achieved when using bridges with slightly decreased donor strength. In the future, such structures could be developed to serve as molecular qubits with optical read-out of the spin-state.

## Author contributions

A. L. carried out the synthesis and characterization of the compounds. A. J. C. K. and S. R. conceptualized the project. M. E. A. supervised the project. R. B., F. J., S. R., and P. T. conducted EPR experiments and analysis. J. Z. and J. S. performed (TD)-DFT calculations. E. S. and M. v. D. crystallized the compounds and conducted XRay crystallography. F. J., M. v. D., S. R., and A. J. C. K. provided supervision, infrastructure, and secured funding. M. E. A., A. L., and A. J. C. K. wrote the first draft and all authors revised and edited the manuscript.

## Conflicts of interest

There are no conflicts to declare.

## Supplementary Material

SC-016-D5SC03673A-s001

SC-016-D5SC03673A-s002

## Data Availability

Crystallographic data for compound 1 has been deposited at the CCDC under 2410919 and can be obtained from https://www.ccdc.cam.ac.uk/data_request/cif. DFT data for this article, including TD-DFT results are available at Zenodo at https://doi.org/10.5281/zenodo.15488688.
