# Supplementary material for: Photostable triphenylmethyl-based diradicals with a degenerate singlet-triplet ground state and strong photoluminescence
Source: Chem Sci. 2025 Jul 7;16(32):14616–24. doi: 10.1039/d5sc03673a (PMC12257165; doi:10.1039/d5sc03673a)
Supplement: SC-016-D5SC03673A-s001 [file SC-016-D5SC03673A-s001.pdf]

Supplementary Information (SI) for Chemical Science.
This journal is © The Royal Society of Chemistry 2025

SUPPORTING INFORMATION

Photostable Triphenylmethyl-Based Diradicals with a Degenerate Singlet-Triplet Ground State and Strong Photoluminescence

Mona E. Arnold,[a] Anika Lebzelter,[a] Philipp Thielert,[b] Rémi Blinder,[c,d] Jonas Schmid,[a] Julia Zolg,[a,d] Emanuele Spatola,[e] Fedor Jelezko,[c,d] Max von Delius,[d,e] Sabine Richert[b‡]* and Alexander J. C. Kuehne[a,d]*

[a]Institute of Organic and Macromolecular Chemistry, Ulm University, Albert-Einstein-Allee 11, 89081 Ulm (Germany).

[b]Institute of Physical Chemistry, University of Freiburg, Albertstraße 21, 79104 Freiburg (Germany).

[c]Institute for Quantum Optics, Ulm University, Albert Einstein Allee 11, 89081 Ulm (Germany).

[d]Center for Integrated Quantum Science and Technology (IQST), Ulm University, Albert-Einstein-Allee 11, 89081 Ulm (Germany).

[e]Institute of Organic Chemistry, Ulm University, Albert-Einstein-Allee 11, 89081 Ulm (Germany).

[‡]current address: Institute of Physical and Theoretical Chemistry, Goethe University Frankfurt, Max-von-Laue-Straße 7, 60438 Frankfurt, Germany.

Contents

1. Materials and Instrumentation ... 3

2. Synthesis ... 4

2.1 Synthesis of Br-TTM ... 4

2.2 Synthesis of 5,7-ICz-HTTM$_2$ ... 6

2.3 Synthesis of 5,7-ICz-TTM$_2$ ... 7

2.4 Synthesis of 5,8-ICz-HTTM$_2$ ... 8

2.5 Synthesis of 5,8-ICz-TTM$_2$ ... 9

2.6 Synthesis of 5,11-ICz-HTTM$_2$ ... 10

2.7 Synthesis of 5,11-ICz-TTM$_2$ ... 11

3. X-Ray Crystallographic Data ... 12

4. Optical Characterization of the Diradicals ... 13

4.1 Concentration-Dependent UV-Vis Absorption Spectra ... 13

4.2 UV-Vis Absorption Spectrum of Crystals of 5,7-ICz-TTM$_2$ ... 14

4.3 Time-Correlated Single Photon Counting ... 15

4.4 Photostability ... 16

5. DFT and TD-DFT Calculations ... 18

5.1 Computational Details ... 18

6. EPR Measurements ... 23

6.1 EPR Setup ... 23

6.2 EPR Characterization of the Diradicals ... 24

6.3 Variable-Temperature EPR evaluation ... 27

6.4 Additional PEANUT data ... 28

7. Mass and NMR Spectra ... 29

1. Materials and Instrumentation

All chemicals are used as commercially available without further purification. Unless otherwise stated, all reactions are performed under argon atmosphere.

NMR spectra are measured by a Bruker Ascend 400/600 spectrometer at 293 K (^{1}H NMR at 400 MHz or 600 MHz; ^{13}C NMR at 101 MHz or 151 MHz). The samples are dissolved in $CDCl_3$, CD_2Cl_2 or DMSO-d_6 which serves as an internal standard. Its chemical shifts δ can be found at δH($CDCl_3$) = 7.26 ppm, δH(CD_2Cl_2) = 5.32 ppm for ^{1}H NMR and at δC($CDCl_3$) = 77.16 ppm, δC(CD_2Cl_2) = 53.84 ppm for ^{13}C NMR.

HRMS (MALDI-FTICR) spectra are performed with a Bruker SolariX containing a DCTB matrix. Chemical ionization (CI) mass spectra are measured with a Thermo Scientific ISQ LT single quadrupole mass spectrometer.

UV-vis spectra are recorded on a Lambda 365 spectrophotometer by Perkin Elmer with UV WinLab as standard software. The samples are diluted in cyclohexane and measured in 1 cm quartz glass cuvettes. The **fluorescence** analysis is performed on a Perkin-Elmer FL 6500 and the **PLQY** is determined with a Hamamatsu Quantaurus-QY (C11347). All optical measurements are performed at room temperature (293 K).

Fluorescence lifetimes are measured by time-correlated single-photon counting (TCPSC) for cyclohexane solutions (10^{-4} M). TCSPC measurements are performed on a custom build laser reflection spectrometer from AIQTEC. A pulsed excitation beam (375 nm, 20 MHz pulse repetition rate) with a picosecond diode laser is used as the light source. The excitation wavelength is removed from the signal by a long-pass filter (400 nm cut-off wavelength). The photoluminescence signal is sent through a spectrograph equipped with a photomultiplier tube which allows for TCSPC measurements in combination with the SPCM data acquisition software. Emission is detected within a wavelength range of 200 nm centered at the respective emission maximum.

2. Synthesis

2.1 Synthesis of Br-TTM

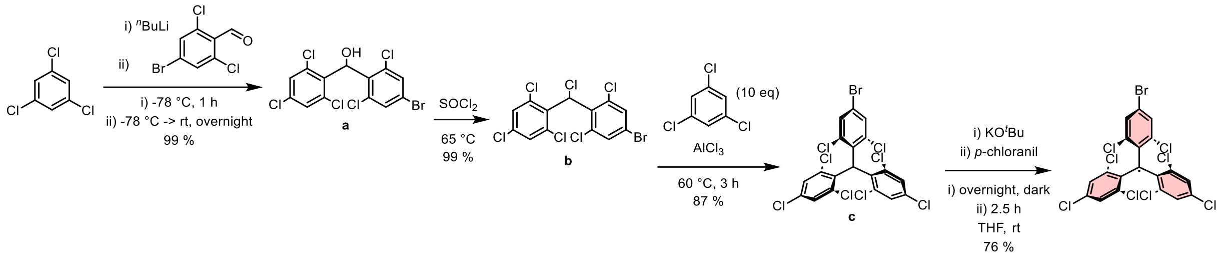

Figure S1: Synthesis of **Br-TTM.** [1]

Br-TTM is synthesized according to the synthesis of (2,6-Dichloro-4-iodophenyl)bis(2,4,6-trichlorophenyl)methyl radical.[1] 7.15 g 1,3,5-trichlorobenzene (39.4 mmol, 1.0 eq.) are dissolved in anhydrous THF at -78 °C and 15.8 mL of a 2.5 M solution of ^{n}BuLi in hexane (39.4 mmol, 1.0 eq.) are added. The mixture is stirred at -78 °C for one hour, after which a solution of 10.0 g 2,6-dichloro-4-bromobenzaldehyde (39.4 mmol, 1.0 eq.) in THF is added quickly. The solution is stirred at -78 °C for 10 minutes and then allowed to warm to room temperature, followed by stirring overnight. The reaction is quenched with water and saturated aqueous ammonium chloride, and the product is then extracted with dichloromethane, yielding (2,6-Dichloro-4-bromophenyl)(2,4,6-trichlorophenyl)methanol (**a**) in quantitative yield. **a** is dissolved in 45 mL $SOCl_2$ and stirred at 65 °C for one hour. After cooling to room temperature, unreacted $SOCl_2$ is hydrolyzed with 1 M HCl solution. DCM is added and the mixture is extracted with brine to yield 1,3,5-trichloro-2-[chloro(2,6-dichloro-4-bromophenyl)methyl]benzene (**b**) in quantitative amount. For the synthesis of Br-HTTM (**c**), 5.13 g of **b** (11.3 mmol, 1.0 eq.) and 21.0 g 1,3,5-trichlorobenzene (116 mmol, 10 eq.) are warmed to 65 °C in a pressure vessel until a homogeneous melt is obtained. 1.62 g $AlCl_3$ (12.1 mmol, 1.1 eq.) are added, and the mixture is stirred for 3 h at 65 °C. After cooling to rt, 1 M hydrochloric acid is added, and the mixture is extracted with DCM. The organic phase is dried over $MgSO_4$, filtrated and concentrated *in vacuo*. The main part of the excess of 1,3,5-trichlorobenzene is sublimated at 85 °C. The crude product is then washed with petroleum ether to remove remaining trichlorobenzene followed by washing with diethyl ether to remove unreacted **b** to yield 5.88 g of Br-HTTM (**c**) (9.82 mmol, 87 %). For the synthesis of Br-TTM, 1.00 g Br-HTTM (1.67 mmol, 1.0 eq.) are dissolved in 150 mL anhydrous THF and 3.77 g KO^{t}Bu (33.6 mmol, 20 eq.) are added and the mixture is stirred overnight at rt. 1.24 g *p*-chloranil (5.05 mmol, 3.0 eq.) are added and the mixture is stirred for further 2.5 h

in the dark. The solvent is removed under reduced pressure and the crude product is purified by silica gel column chromatography using PE:DCM (5:1) as an eluent. The received red fraction is resolved in THF and the procedure is repeated with further 2.45 g KO^{t}Bu (21.9 mmol, 20 eq.) and 1.24 g *p*-chloranil (5.04 mmol, 3.0 eq.). After purification, 0.767 g Br-TTM (1.28 mmol, 76 %) are obtained.

a:

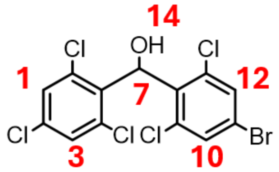

^{1}H NMR (600 MHz, $CDCl_3$, 293 K): δ = 7.47 (s, 2 H, **10/12**), 7.33 (s, 2 H, **1/3**), 6.70 (d, $^3J_{H\text{-}H}$ = 10.5 Hz, 1 H, **7**), 3.34 (d, $^3J_{H\text{-}H}$ = 10.6 Hz, 1 H, **14**) ppm.

^{13}C{^{1}H} NMR (151 MHz, $CDCl_3$, 293 K): δ = 136.1, 135.9, 134.9, 134.5, 134.4, 132.4, 129.7, 121.9, 73.0 ppm.

MS (CI): *m z*$^{-1}$ = calc. for $C_{13}H_5BrCl_5$ $[M{-}OH]^+$: 418, found: 418.

b:

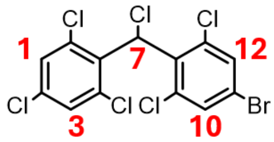

^{1}H NMR (600 MHz, $CDCl_3$, 293 K): δ = 7.50 (s, 2 H, **10/12**), 7.36 (s, 2 H, **1/3**), 7.00 (s, 1 H, **7**) ppm.

^{13}C{^{1}H} NMR (151 MHz, $CDCl_3$, 293 K): δ = 137.0, 136.9, 135.0, 132.7, 132.6, 132.1, 129.9, 122.5, 55.5 ppm.

MS (CI): *m z*$^{-1}$ = calc. for $C_{13}H_5BrCl_5$ $[M{-}Cl]^+$: 418, found: 418.

Br-HTTM:

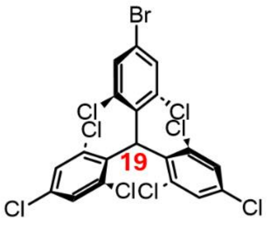

^{1}H NMR (400 MHz, $CDCl_3$, 293 K): δ = 7.50 (d, $^4J_{H\text{-}H}$ = 2.1 Hz, 1 H), 7.38 (d, $^4J_{H\text{-}H}$ = 2.0 Hz, 1 H), 7.36 (d, $^4J_{H\text{-}H}$ = 2.3 Hz, 2 H), 7.23 (d, $^4J_{H\text{-}H}$ = 2.3 Hz, 2 H), 6.66 (s, 1 H, **19**) ppm.

MS (CI): *m z*$^{-1}$ = calc. for $C_{19}H_7BrCl_7$ $[M{-}Cl]^+$: 563, found: 563.

Br-TTM: **MS (CI)**: *m z*$^{-1}$ = calc. for $C_{19}H_6BrCl_8^{\bullet}$: 596, found: 596.

5 mg Br-TTM dissolved in 0.6 mL $CDCl_3$ only shows solvent signals in the ^{1}H NMR (400 MHz, 293 K), proving the absence of unreacted Br-HTTM (s. Figure S45).

2.2 Synthesis of 5,7-ICz-HTTM₂

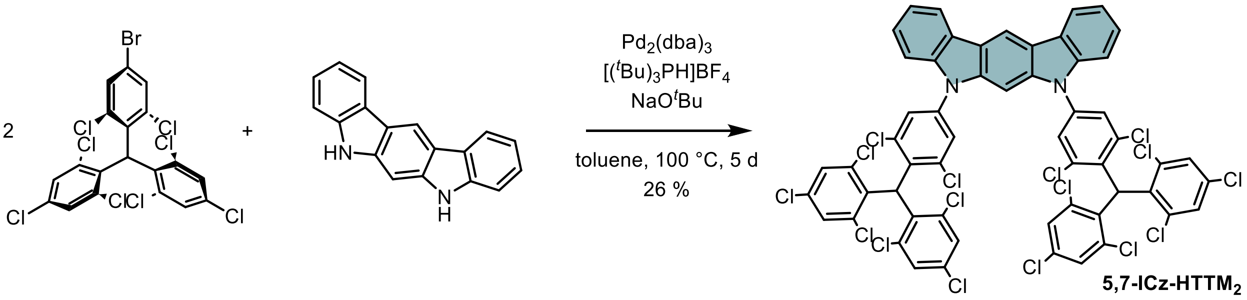

Figure S2: Synthesis of **5,7-ICz-HTTM₂**.

201 mg Br-HTTM (0.336 mmol, 2.2 eq.), 39 mg 5,7-dihydroindolo[2,3-*b*]carbazole (0.151 mmol, 1.0 eq.), 23 mg [(^{t}Bu)$_3$PH]BF_4 (0.078 mmol, 0.5 eq.) and 57 mg NaO^{t}Bu (0.593 mmol, 4.0 eq.) are dissolved in 60 mL anhydrous toluene. After the addition of 35 mg $Pd_2(dba)_3$ (0.038 mmol, 0.25 eq.), the reaction mixture is warmed to 100 °C and stirred for five days in the dark. The mixture is allowed to cool to rt and is extracted with brine and DCM. The organic phase is collected, dried over $MgSO_4$ and the solvent is evaporated under reduced pressure. The crude product is purified by column chromatography with 5:1 PE:DCM as the eluent resulting in 51 mg **5,7-ICz-HTTM₂** as a white solid (0.040 mmol, 26 %).

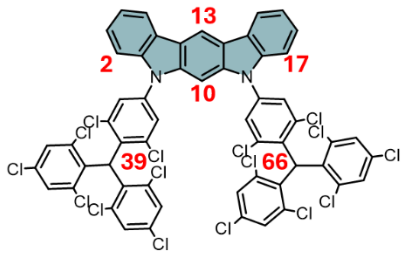

^{1}H NMR (600 MHz, CD_2Cl_2, 293 K): δ = 8.82 (s, 1 H, **13**), 8.24 (d, 2 H, **2/17**), 7.72 (d, $^3J_{H-H}$ = 2.3 Hz, 1 H), 7.66 (d, $^3J_{H-H}$ = 2.3 Hz, 1 H), 7.53 (d, $^3J_{H-H}$ = 2.1 Hz, 1 H), 7.52 (s, 1 H), 7.48 - 7.39 (m, 6 H), 7.39 – 7.35 (m, 2H), 7.34 (d, $^4J_{H-H}$ = 2.3 Hz, 1 H), 7.32 (d, $^4J_{H-H}$ = 2.5 Hz, 1 H), 7.19 (bs, 1 H, **10**), 6.87 (s, 2 H, **39/66**) ppm.

^{13}C{^{1}H} NMR (151 MHz, CD_2Cl_2, 293 K): δ = 141.2, 141.1, 140.7, 140.5, 139.0, 138.9, 138.6, 138.5, 138.4, 138.3, 138.2, 138.1, 137.7, 137.7, 137.6, 137.6, 134.6, 134.5, 134.5, 134.5, 134.2, 134.2, 134.2, 134.1, 134.0, 130.7, 130.5, 130.5, 129.4, 129.0, 128.9, 128.9, 128.6, 128.5, 127.9,

126.9, 126.3, 126.1, 126.0, 125.6, 124.7, 124.5, 121.5, 121.3, 120.3, 120.3, 120.0, 119.8, 112.2, 110.0, 109.8, 89.9, 89.5, 50.5 ppm.

The two signal sets observed in the NMR spectrum correspond to the diastereomers arising from the chirality of the two HTTM moieties (see Figure S46).

HR-MS (MALDI-FTICR): *m* z^{-1} = calc. for $C_{56}H_{24}Cl_{16}N_2$: 1291.6847, found: 1291.6851; $\delta m/m$ = 0.3 ppm.

2.3 Synthesis of 5,7-ICz-TTM$_2$

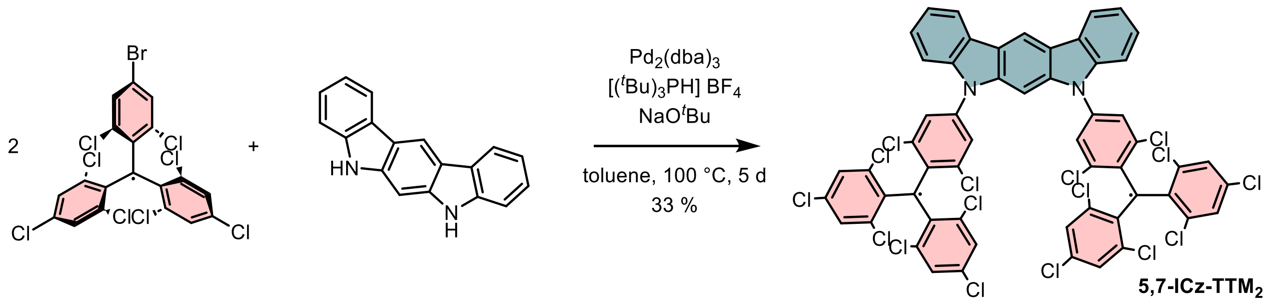

Figure S3: Synthesis of **5,7-ICz-TTM$_2$**.

295 mg Br-TTM (0.494 mmol, 2.2 eq.), 58 mg 5,7-dihydroindolo[2,3-*b*]carbazole (0.225 mmol, 1.0 eq.), 34 mg [(^{t}Bu)$_3$PH]BF$_4$ (0.118 mmol, 0.52 eq.) and 98 mg NaO^{t}Bu (1.020 mmol, 4.6 eq.) are dissolved in 110 mL anhydrous toluene. After the addition of 55 mg $Pd_2(dba)_3$ (0.060 mmol, 0.27 eq.), the reaction mixture is warmed to 100 °C and stirred for five days in the dark. After cooling to rt, 112 mg *p*-chloranil (0.456 mmol, 2 eq.) are added and the mixture is stirred for further two hours at rt. 96 mg of a dark green solid (0.075 mmol, 33 %) are obtained after column chromatography with 5:1 → 4:1 PE:DCM eluent gradient.

HR-MS (MALDI-FTICR): *m* z^{-1} = calc. for $C_{56}H_{22}Cl_{16}N_2^{2\bullet}$: 1289.6691, found: 1289.6667; $\delta m/m$ = 1.8 ppm.

2.4 Synthesis of 5,8-ICz-HTTM$_2$

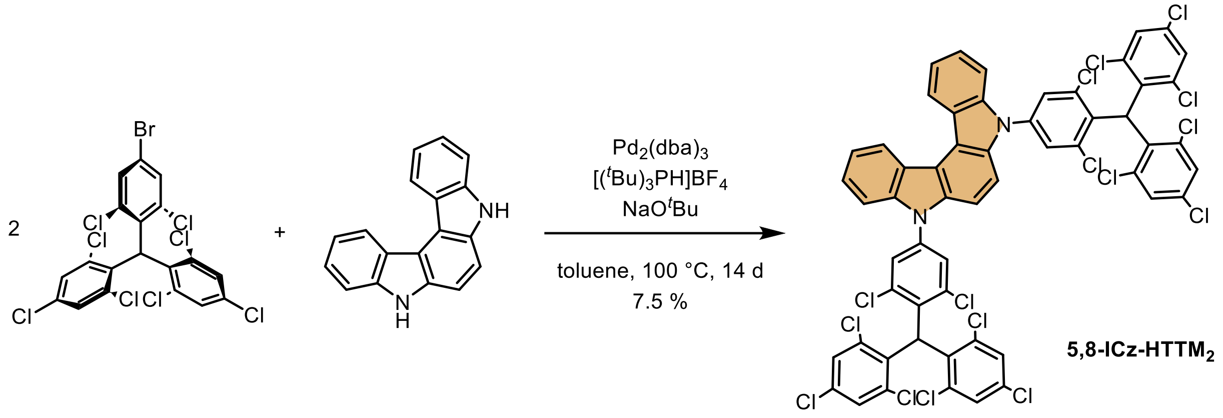

Figure S4: Synthesis of **5,8-ICz-HTTM$_2$**.

211 mg Br-HTTM (0.353 mmol, 2.3 eq.), 39 mg 5,8-dihydroindolo[2,3-*b*]carbazole (0.151 mmol, 1.0 eq.), 28 mg [(^{t}Bu)$_3$PH]BF$_4$ (0.096 mmol, 0.64 eq.) and 56 mg NaO^{t}Bu (0.585 mmol, 3.9 eq.) are dissolved in 100 mL anhydrous toluene. After adding 39 mg Pd$_2$(dba)$_3$ (0.042 mmol, 0.28 eq.), the reaction mixture is warmed to 100 °C and stirred for 14 days in the dark. The mixture is allowed to cool to rt and is extracted with brine and DCM. The organic phase is collected, dried over $MgSO_4$ and the solvent is evaporated under reduced pressure. The crude product is subjected to column chromatography using a 5:1 PE:DCM eluent, yielding 15 mg of a **5,8-ICz-HTTM$_2$** as a white solid (0.011 mmol, 7.5 %).

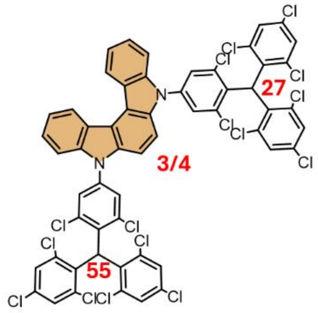

^{1}H NMR (600 MHz, CD_2Cl_2, 293 K): δ = 8.93 (d, $^3J_{H\text{-}H}$ = 8.0 Hz, 2 H, **3/4**), 7.69 (d, $^3J_{H\text{-}H}$ = 2.3 Hz, 2 H), 7.57 – 7.54 (m, 8 H), 7.51 (m, 2 H), 7.48 (d, $^3J_{H\text{-}H}$ = 2.3 Hz, 2 H), 7.46 (d, $^3J_{H\text{-}H}$ = 2.3 Hz, 2 H), 7.36 (d, $^3J_{H\text{-}H}$ = 2.3 Hz, 2 H), 7.32 (d, $^3J_{H\text{-}H}$ = 2.3 Hz, 2 H), 6.90 (s, 2 H, **27/55**) ppm.

^{13}C{^{1}H} NMR (151 MHz, CD_2Cl_2, 293 K): δ = 140.9, 138.9, 138.5, 138.4, 138.1, 137.7, 137.6, 136.8, 134.9, 134.5, 134.5, 134.3, 130.6, 130.5, 129.6, 129.0, 128.9, 128.0, 126.3, 124.0, 123.6, 120.9, 117.8, 110.2, 109.3, 50.6 ppm.

HR-MS (MALDI-FTICR): $m\ z^{-1}$ = calc. for $C_{56}H_{24}Cl_{16}N_2$: 1291.6847, found: 1291.6820; $\delta m/m$ = 2.1 ppm.

2.5 Synthesis of 5,8-ICz-TTM$_2$

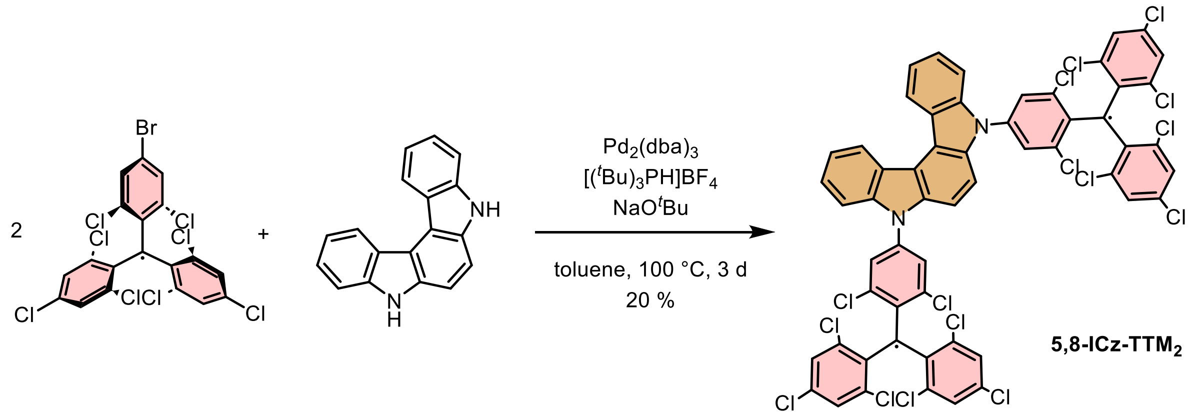

Figure S5: Synthesis of **5,8-ICz-TTM$_2$**.

200 mg Br-TTM (0.335 mmol, 2.2 eq.), 39 mg 5,8-dihydroindolo[2,3-*b*]carbazole (0.152 mmol, 1.0 eq.), 22 mg $[(^tBu)_3PH]BF_4$ (0.076 mmol, 0.50 eq.) and 55 mg NaO^tBu (0.574 mmol, 3.8 eq.) are solved in 100 mL anhydrous toluene. 35 mg $Pd_2(dba)_3$ (0.038 mmol, 0.25 eq.) are added, and the reaction mixture is stirred at 100 °C for three days in the dark. After cooling to rt, the mixture is extracted with brine and DCM and the organic phase is dried over $MgSO_4$ and the solvent is evaporated under reduced pressure. The crude product is purified by column chromatography with a PE:DCM eluent gradient (5:1 → 4:1), yielding 38 mg of **5,8-ICz-TTM$_2$** as a green solid (0.030 mmol, 20 %).

HR-MS (MALDI-FTICR): $m\ z^{-1}$ = calc. for $C_{56}H_{22}Cl_{16}N_2^{2\bullet}$: 1289.6691, found: 1289.6667; $\delta m/m$ = 1.8 ppm.

2.6 Synthesis of 5,11-ICz-HTTM$_2$

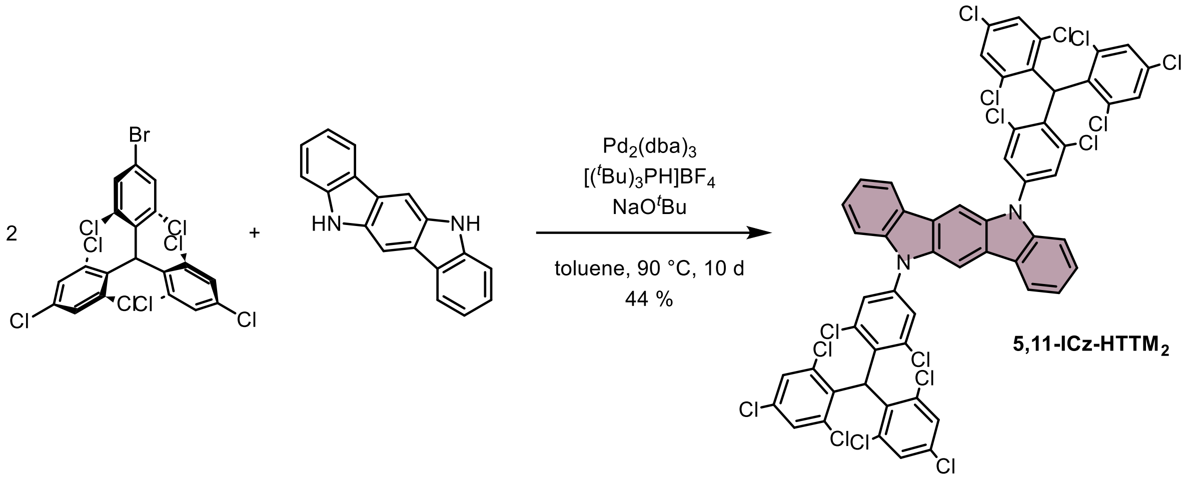

Figure S6: Synthesis of **5,11-ICz-HTTM$_2$**.

200 mg Br-HTTM (0.334 mmol, 2.3 eq.), 37 mg 5,11-dihydroindolo[3,2-*b*]carbazole (0.146 mmol, 1.0 eq.), 23 mg $[(^tBu)_3PH]BF_4$ (0.079 mmol, 0.54 eq.) and 57 mg NaO^tBu (0.589 mmol, 4.0 eq.) are dissolved in 100 mL anhydrous toluene. After adding 35 mg $Pd_2(dba)_3$ (0.039 mmol, 0.26 eq.), the reaction mixture is warmed to 90 °C and stirred for ten days in the dark. After cooling to rt, the mixture is extracted with brine and DCM and the organic phase is collected, dried over $MgSO_4$ and the volatiles are evaporated under reduced pressure. For purification, column chromatography of the crude product is performed with 4:1 PE:DCM as an eluent. The collected fractions are washed twice with pentane resulting in 82 mg of **5,11-ICz-HTTM$_2$** as a white solid (0.063 mmol, 44 %).

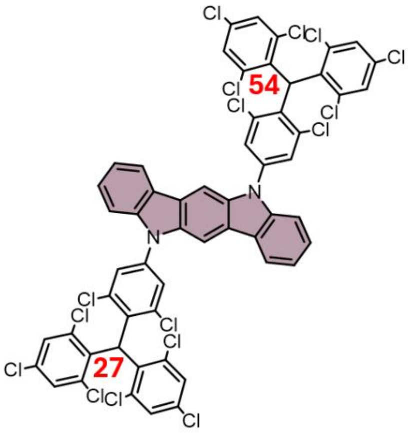

^{1}H NMR (400 MHz, $CDCl_3$, 293 K): δ = 8.19 (d, $^3J_{H\text{-}H}$ = 7.7 Hz, 2 H), 8.04 (s, 2 H), 7.72 (d, $^3J_{H\text{-}H}$ = 2.2 Hz, 2 H), 7.59 (d, $^3J_{H\text{-}H}$ = 2.3 Hz, 2 H), 7.52 – 7.38 (m, 8 H), 7.37 – 7.28 (m, 6 H), 6.91 (s, 2 H, **27/54**) ppm.

$^{13}C\{^1H\}$ NMR (151 MHz, $CDCl_3$, 293 K): δ = 141.5, 138.8, 138.6, 138.2, 137.9, 137.5, 136.6, 134.3, 134.0, 130.4, 130.3, 128.7, 128.5, 126.9, 126.8, 124.0, 123.9, 120.8, 120.6, 109.6, 100.2, 50.3 ppm.

HR-MS (MALDI-FTICR): $m\ z^{-1}$ = calc. for $C_{56}H_{24}Cl_{16}N_2$: 1291.6847, found: 1291.6837; $\delta m/m$ = 0.8 ppm.

2.7 Synthesis of 5,11-ICz-TTM$_2$

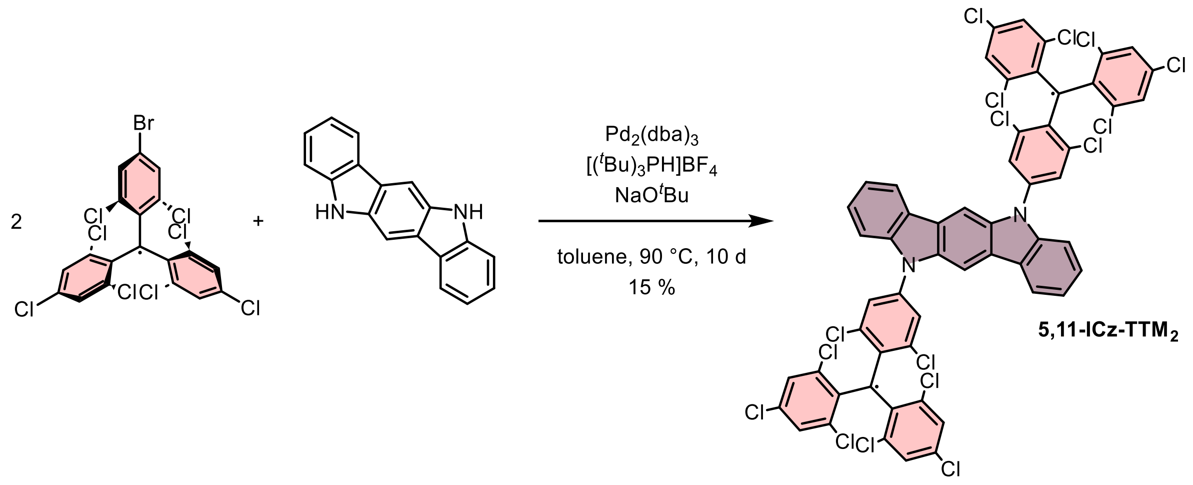

Figure S7: Synthesis of **5,11-ICz-TTM$_2$**.

249 mg Br-TTM (0.416 mmol, 2.3 eq.), 47 mg 5,11-dihydroindolo[3,2-*b*]carbazole (0.182 mmol, 1.0 eq.), 27 mg $[(^tBu)_3PH]BF_4$ (0.091 mmol, 0.50 eq.) and 70 mg NaO^tBu (0.728 mmol, 4.0 eq.) are dissolved in 60 mL anhydrous toluene. After the addition of 44 mg $Pd_2(dba)_3$ (0.048 mmol, 0.26 eq.), the reaction mixture is warmed to 90 °C and stirred for ten days in the dark. The mixture is allowed to cool to rt and extracted with brine and DCM. The organic phase is dried over $MgSO_4$ and the solvent is evaporated under reduced pressure. The crude product is purified by column chromatography with 4:1 PE:DCM as the eluent, resulting in 35 mg of a green solid (27 mmol, 15 %).

HR-MS (MALDI-FTICR): $m\ z^{-1}$ = calc. for $C_{56}H_{22}Cl_{16}N_2^{2\bullet}$: 1289.6691, found: 1289.6683; $\delta m/m$ = 0.6 ppm.

3. X-Ray Crystallographic Data

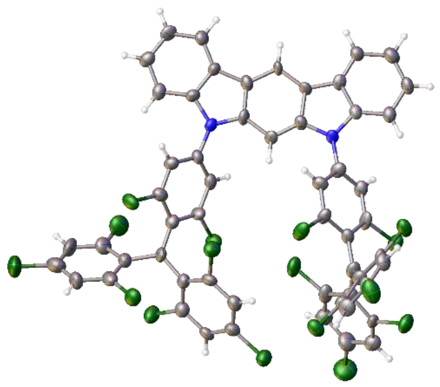

Table S1: Results of crystallographic analysis of **5,7-ICz-TTM$_2$**

**Empirical formula**	$C_{56}H_{22}Cl_{16}N_2$
**Formula weight**	1409.73
***T* / K**	150.00(14)
**Crystal system**	triclinic
**Space group**	P-1
***a* / Å**	8.2856(12)
***b* / Å**	17.898(2)
***c* / Å**	21.953(3)
***α* / °**	108.813(11)
***β* / °**	97.313(12)
***γ* / °**	93.276(11)
***V* / Å^3**	3039.7(7)
**Z**	2
**ρ_{calc} / g cm^{-3}**	1.540
**μ / mm^{-1}**	6.978
***F*(000)**	1418.0
**Crystal size / mm^3**	0.26 × 0.07 × 0.03
**Radiation**	Cu Kα (λ = 1.54184)
**2*Θ* range for data collection / °**	7.818 to 146.562
**Index ranges**	$-10 \le h \le 7$, $-22 \le k \le 21$, $-23 \le l \le 27$
**Reflections collected**	22156
**Independent reflections**	11740 [R_{int} = 0.1930, R_{sigma} = 0.2999]
**Data / restraints / parameters**	11740/0/667
**Goodness-of-fit on F^2**	0.852
**Final *R* indexes [I>=2σ (*I*)]**	R_1 = 0.0882, wR_2 = 0.1854
**Final *R* indexes [all data]**	R_1 = 0.1899, wR_2 = 0.2727
**Largest diff. peak / hole / e Å^{-3}**	0.59/-0.46

5,7-ICz-TTM$_2$ was crystallized by solvent diffusion of ^{n}hexane into a solution of **5,7-ICz-TTM$_2$** in toluene.

CCDC number 2410919 contains the supplementary crystallographic data for this paper. These data can be obtained free of charge from The Cambridge Crystallographic Data Centre *via* www.ccdc.cam.ac.uk/data_request/cif.

4. Optical Characterization of the Diradicals

Table S2: Absorption maxima (λ_{abs}) and photoluminescence quantum yields ϕ for this work and related donor-functionalized diradicals in the literature. λ_{abs} and ϕ were measured in cyclohexane or toluene solutions. [2–6]

compound	λ_{abs} / nm	ϕ / %	reference
**5,7-ICz-TTM$_2$**	641	18	this work
**5,8-ICz-TTM$_2$**	661	2	this work
**5,11-ICz-TTM$_2$**	674	2	this work
**DR1**	600	16	[2]
**Cz$_4$-TTM$_2$**	663	2.8	[3]
**(TTM-Cz)$_2$-An**	617	3	[4]
**TTM-*m*Ph-TTM**	576	0.6	[5]
**(M$_2$-TTM)$_2$-CDT**	756	0.8	[6]

4.1 Concentration-Dependent UV-Vis Absorption Spectra

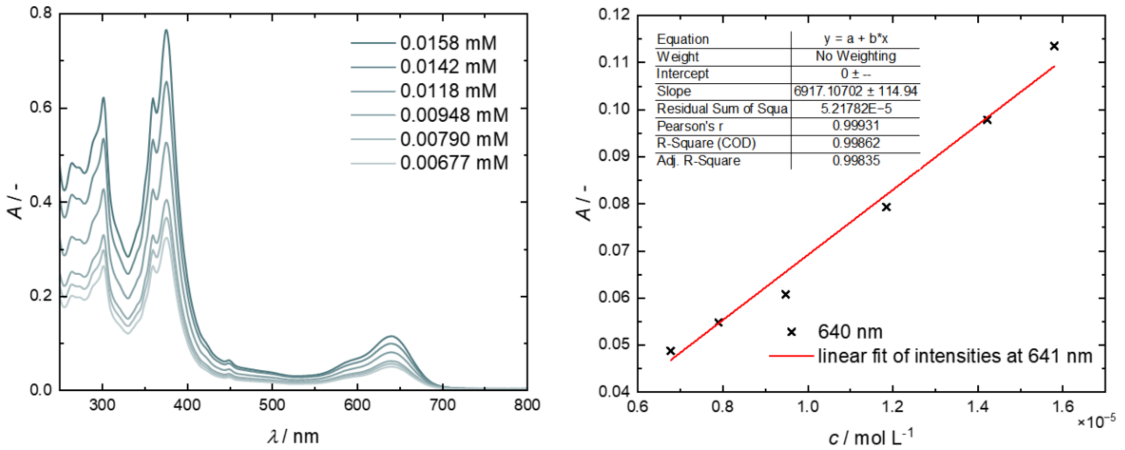

Figure S8: Left: Concentration-dependent absorption spectra of **5,7-ICz-TTM$_2$**, measured for CH solution at 293 K. Right: Linear fitting of absorption at 641 nm.

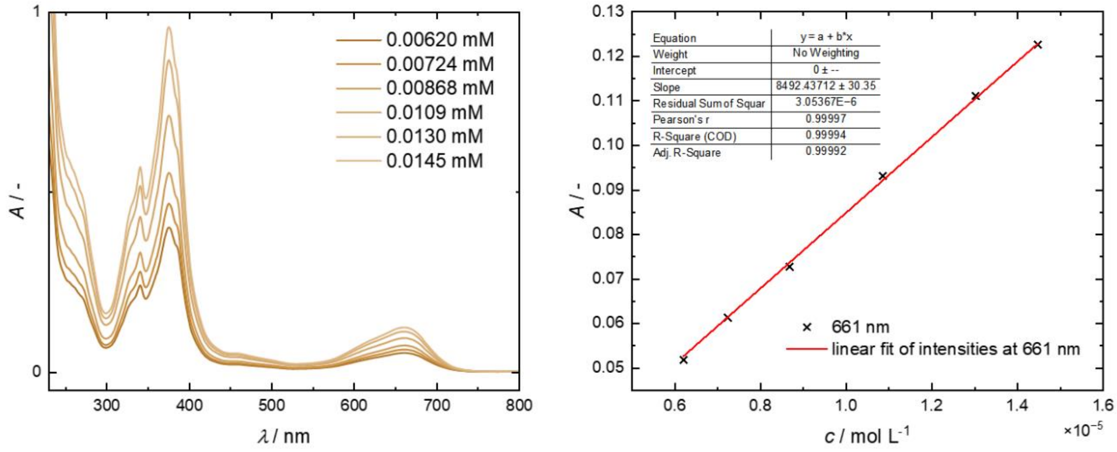

Figure S9: Left: Concentration-dependent absorption spectra of **5,8-ICz-TTM$_2$**, measured in CH at 293 K. Right: Linear fitting of absorption at 661 nm.

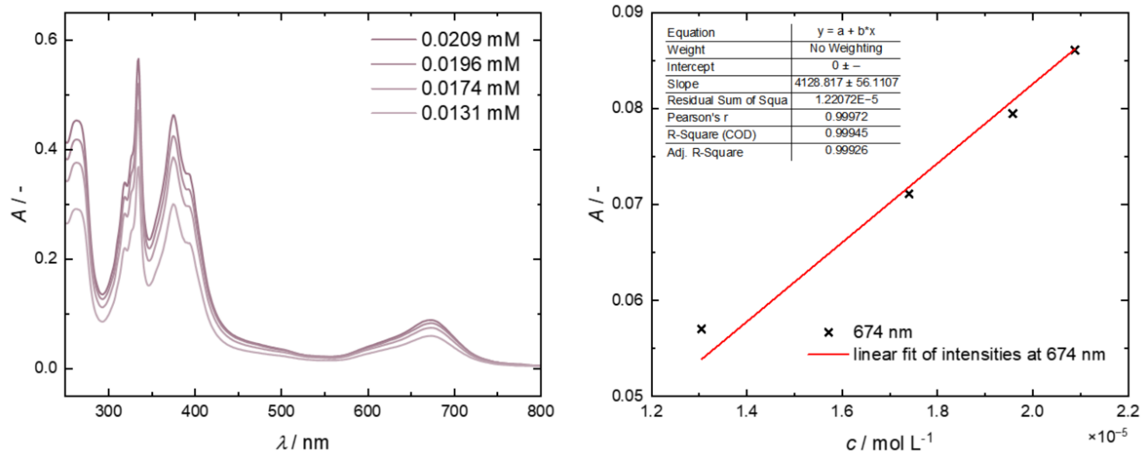

Figure S10: Left: Concentration-dependent absorption spectra of **5,11-ICz-TTM$_2$** measured in CH at 293 K. Right: Linear fitting of absorption at 674 nm.

4.2 UV-Vis Absorption Spectrum of Crystals of 5,7-ICz-TTM$_2$

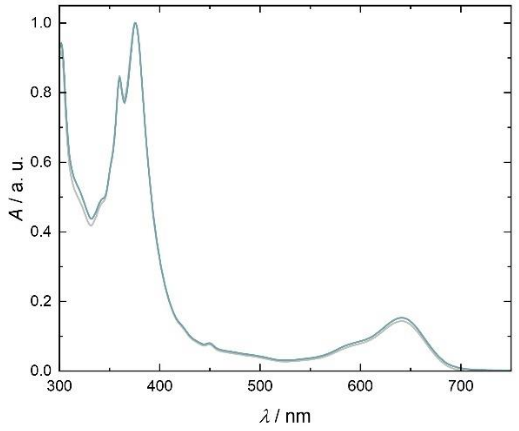

Figure S11: Absorption Spectrum of **5,7-ICz-TTM$_2$** in cyclohexane, before (—) and after (—) crystallization.

4.3 Time-Correlated Single Photon Counting

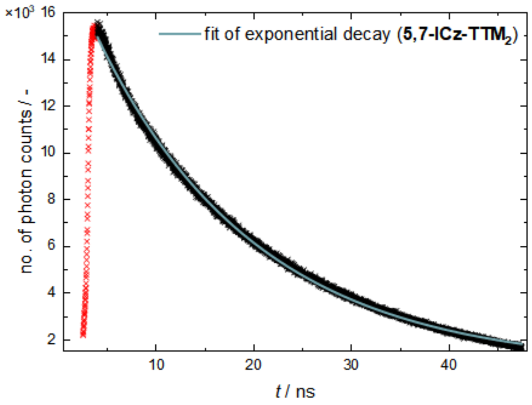

Figure S12: Fluorescence decay of **5,7-ICz-TTM₂** in CH at 293 K after excitation at 375 nm, including the fit of exponential decay (blue line).

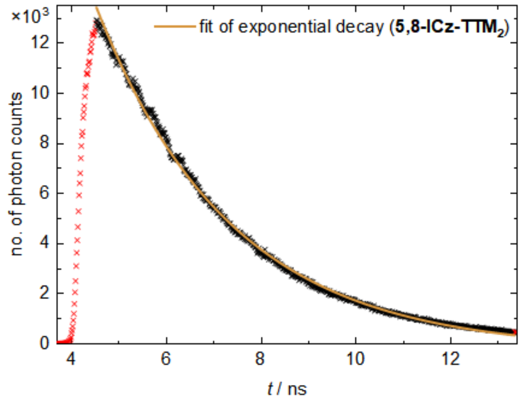

Figure S13: Fluorescence decay of **5,8-ICz-TTM₂** in CH at 293 K after excitation at 375 nm, including the fit of exponential decay (orange line).

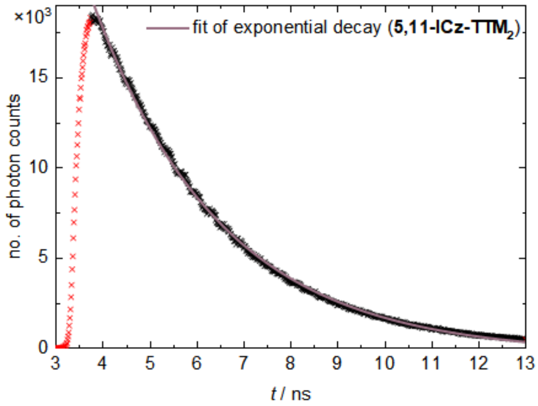

Figure S14: Fluorescence decay of **5,11-ICz-TTM₂** in CH at 293 K after excitation at 375 nm, including the fit of exponential decay (purple line).

4.4 Photostability

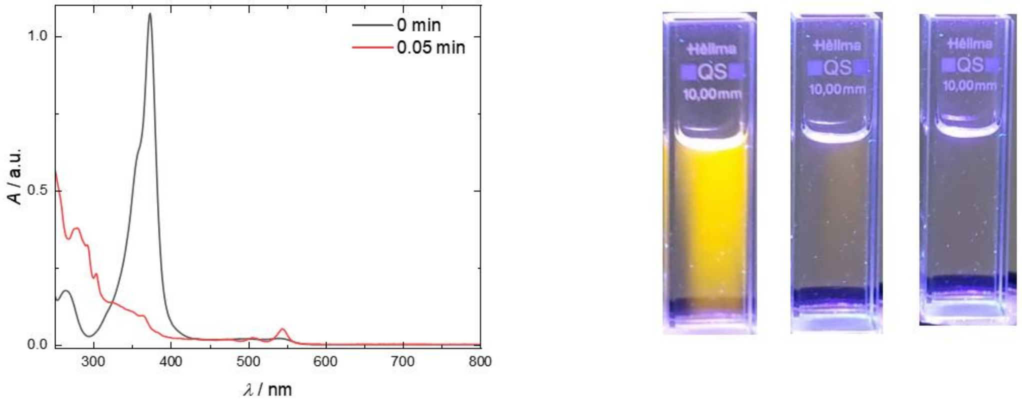

a **b**

Figure S15: Photostability measurement of TTM. **a)** Absorption spectra after illumination (λ_{ex} = 365 nm, P = 5 W). **b)** Visualization of change of emission in dependance of time – left: 0 min, middle: 0.03 min, right: 0.05 min.

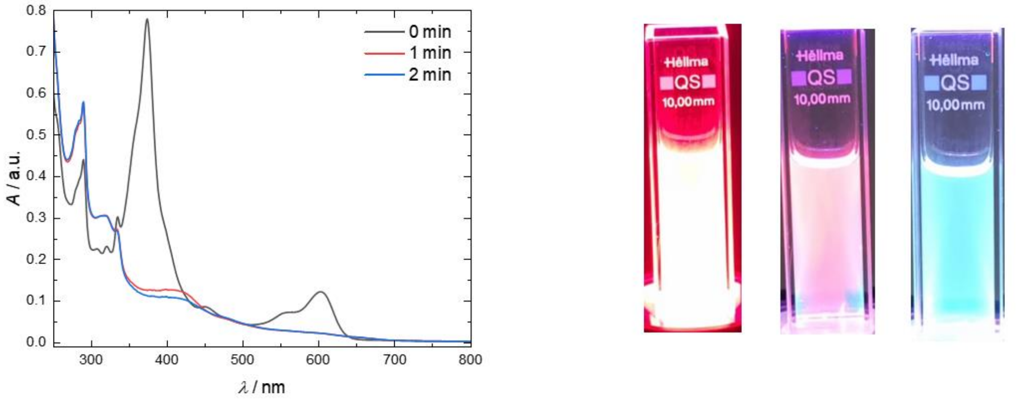

a **b**

Figure S16: Photostability measurement of **TTM-Cz. a)** Absorption spectra after illumination (λ_{ex} = 365 nm, P = 5 W). **b)** Visualization of change of emission in dependance of time – left: 0 min, middle: 0.5 min, right: 2 min.

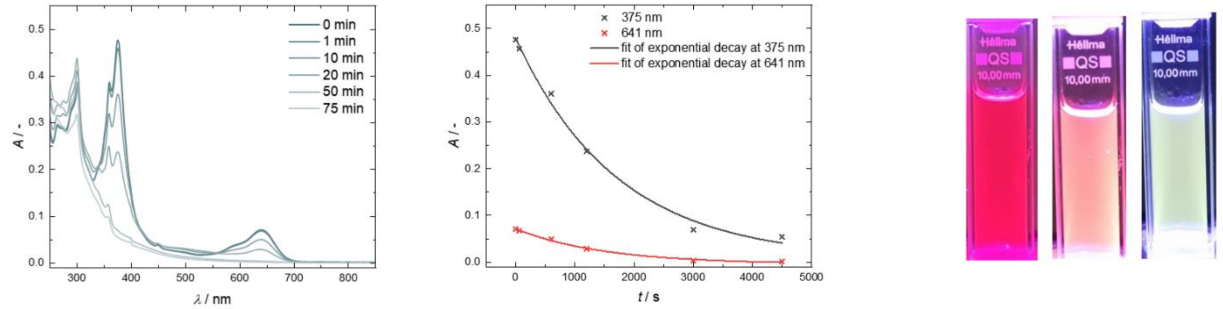

a **b** **c**

Figure S17: Photostability measurement of **5,7-ICz-TTM₂. a)** Absorption spectra after illumination (λ_{ex} = 365 nm, P = 5 W). **b)** Absorption maxima plotted *versus* time with fit of exponential decay (solid lines). **c)** Visualization of change of emission in dependance of time – left: 0 min, middle: 20 min, right: 75 min.

The half-life was determined by fitting the data with a mono-exponential decay function. **5,7-ICz-TTM₂** has $t_{\frac{1}{2}} = 19\ \mathrm{min}.$

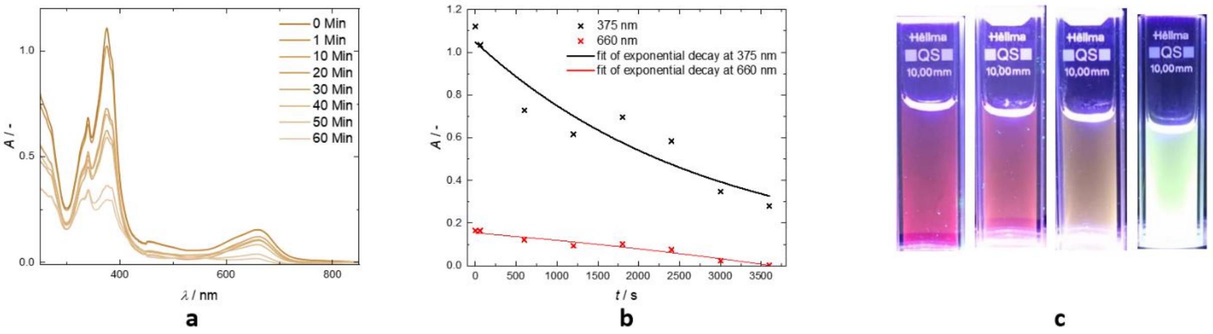

Figure S18: Photostability measurement of **5,8-ICz-TTM₂. a)** Absorption spectra after illumination (λ_{ex} = 365 nm, P = 5 W). **b)** Absorption maxima plotted *versus* time. **c)** Visualization of change of emission in dependance of time – left: 0 min, middle left: 20 min, middle right: 30 min, right: 60 min.

Half-life **5,8-ICz-TTM₂** received from fitting with a mono-exponential decay: $t_{\frac{1}{2}} = 34\ \mathrm{min}.$

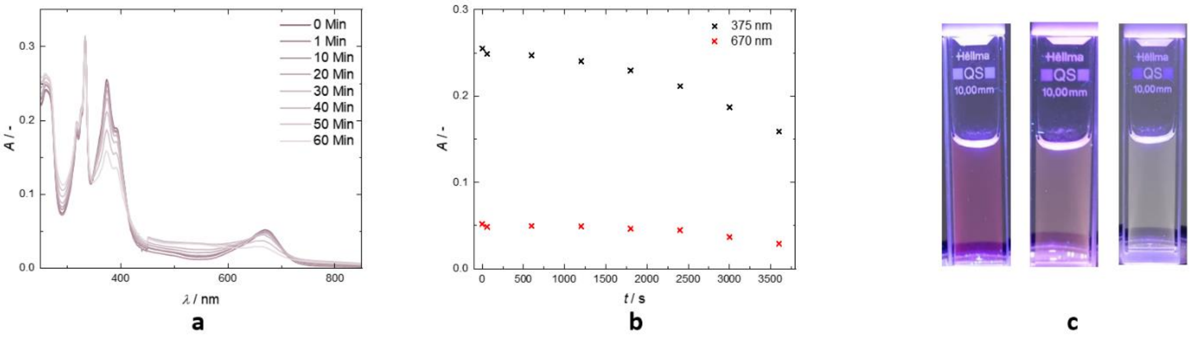

Figure S19: Photostability measurement of **5,11-ICz-TTM₂**. **a)** Absorption spectrum after illumination (λ_{ex} = 365 nm, P = 5 W) at different time points. **b)** Absorption maxima plotted *versus* time. **c)** Visualization of change of emission in dependance of time – left: 0 min, middle: 20 min, right: 60 min.

The photodegradation data of **5,11-ICz-TTM₂** cannot be fitted with a mono-exponential decay function after irradiation for 1 hour. The absorption of the sample has not even decayed to half of the starting value, which is why we can derive that the half-life must be longer than 60 minutes.

5. DFT and TD-DFT Calculations

5.1 Computational Details

Density functional theory (DFT) computations of the diradicals are performed with Orca (version 6.0.0). [7,8] Geometry optimizations are performed using the M06-L [9] functional and a def2-SVP [10,11] basis set with a Grimme D3 [12] dispersion correction and a CPCM solvation model [13] to simulate a cyclohexane environment. The structures are assumed to be fully converged if all frequencies are higher than 100 cm^{-1}.

Single-point calculations are performed on the converged structures using the PBE0[14] functional and a def2-TZVP basis set with a Grimme D3 dispersion correction and a SMD [15] solvent model to simulate a cyclohexane environment.

Geometry optimizations and single-point calculations are performed for the open-shell singlet and the open-shell triplet state. For the open-shell singlet state, a broken-symmetry (BS) approach is used.

Time dependent density functional theory (TD-DFT) calculations are performed on the converged geometries using the PBE0 functional and a def2-TZVP basis set with a Grimme D3 dispersion correction and a CPCM solvation model to simulate a cyclohexane environment. The first 50 excited states are calculated, and a natural transition orbital (NTO) analysis is performed for the first 4 excited states. All calculated absorption spectra are plotted with Orca using gaussian functions with a full width at half maximum (FWHM) of 1000 cm^{-1}.

DFT calculations of the isolated IdCz linkers, saturated with hydrogen, are performed with Gaussian g16.C.01. [16] The structures are optimized *in vacuo*, employing the restricted PBE0 functional is applied with the 6-31+G(d) basis set. [17,18] . The ionization energies *IE* are obtained as the difference in total energy of the neutral and cationic species. The energy of the cations is calculated at the unrestricted PBE0 level with the above-described basis sets by using the optimized neutral structures without re-optimization.

Table S3: Experimentally and theoretically determined photophysical properties of the diradicals. Experimentally determined absorption maxima were measured in cyclohexane solutions (10^{-4} M). Theoretically determined absorption maxima for the broken symmetry (BS) singlet and the triplet state (T) were calculated at PBE0-GD3/def2-TZVP level of theory with a CPCM solvation model to simulate a cyclohexane environment. HOMO Energies and ionization energies *IE* of the isolated ICz donors calculated at the PBE0/6-31+G(d) level of theory (*in vacuo*).

Compound	*E*(HOMO) / eV	*IE* / eV	$\lambda_{\mathbf{abs,exp}}$ / nm	$\lambda_{\mathbf{abs,theo}}$ (BS)/ nm	$\lambda_{\mathbf{abs,theo}}$ (T)/ nm
**5,7-ICz-TTM$_2$**	−5.52	6.80	641	660, 598	640
**5,8-ICz-TTM$_2$**	−5.43	6.76	661	662	643
**5,11-ICz-TTM$_2$**	−5.40	6.70	674	673	653

5.2 Simulated Absorption Spectra

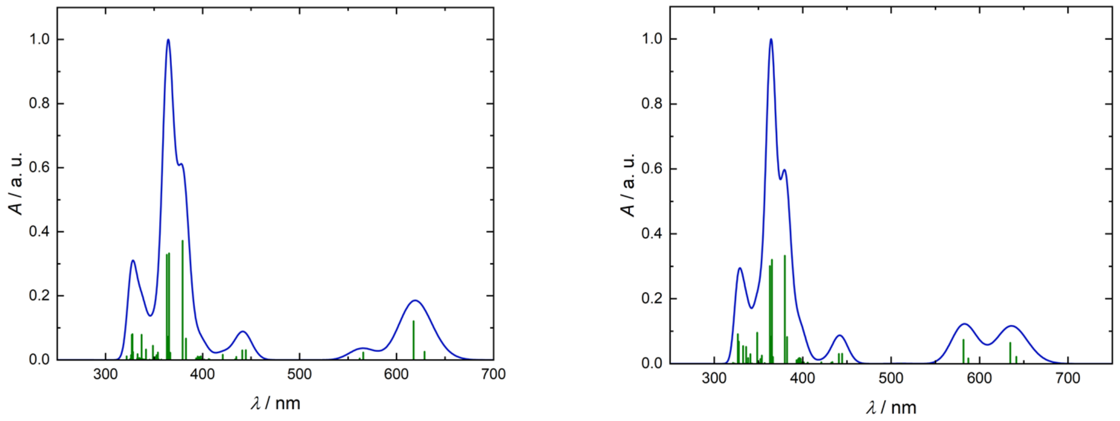

Figure S20: Simulated absorption spectra of *(P,P)*-**5,7-ICz-TTM₂** including oscillator strengths (green) at the PBE0/def2--TZVP, GD3 level of theory. Left: Triplet, right: Broken-symmetry singlet.

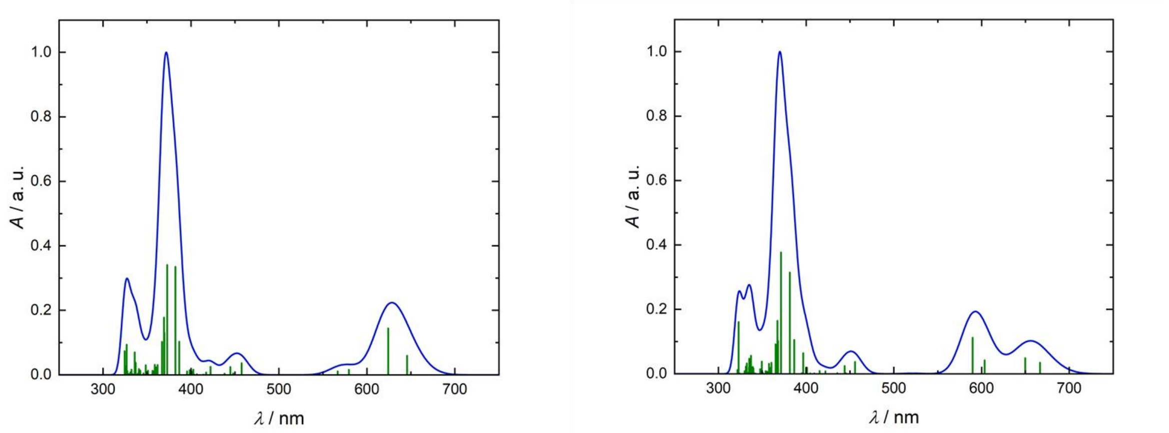

Figure S21: Simulated absorption spectra of *(P,M)*-**5,7-ICz-TTM₂** including oscillator strengths (green) at the PBE0/def2-TZVP, GD3 level of theory. Left: Triplet, right: Broken-symmetry singlet.

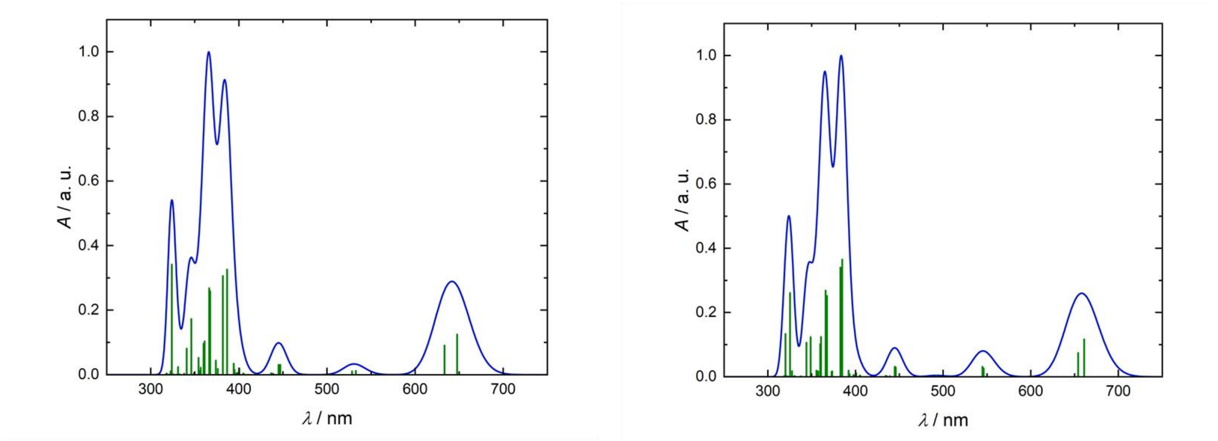

Figure S22: Simulated absorption spectra of **5,8-ICz-TTM₂** including oscillator strengths (green) at the PBE0/def2-TZVP, GD3 level of theory. Left: Triplet, right: Broken-symmetry singlet.

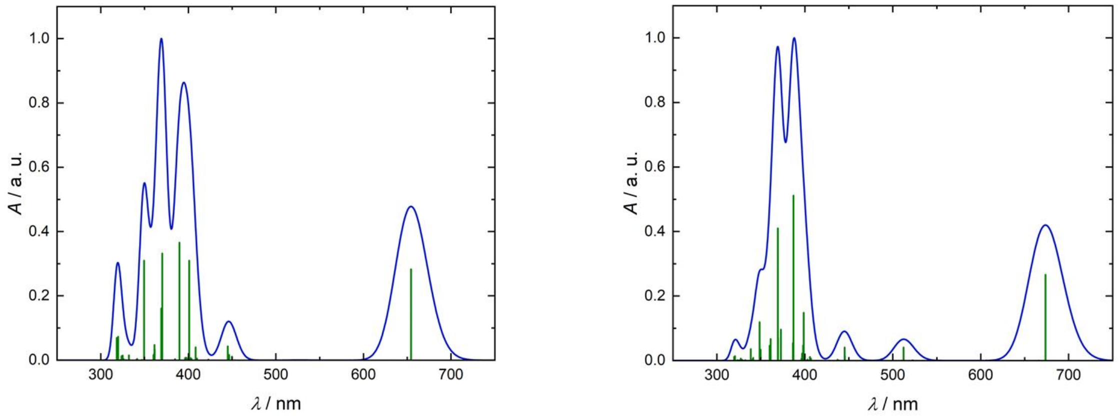

Figure S23: Simulated absorption spectra of **5,11-ICz-TTM₂** including oscillator strengths (green) at the PBE0/def2-TZVP, GD3 level of theory. Left: Triplet, right: Broken-symmetry singlet.

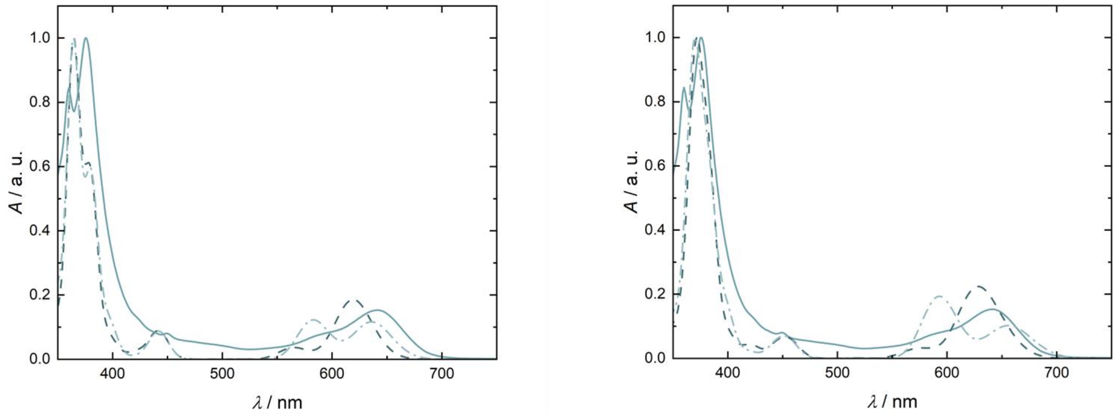

Figure S24: Experimental (solid lines) and calculated absorption spectra of the triplet (dashed lines) and open-shell singlet (dashed dotted lines) state of *(P,P)*-**5,7-ICz-TTM₂** (left) and *(P,M)*-**5,7-ICz-TTM₂** (right).

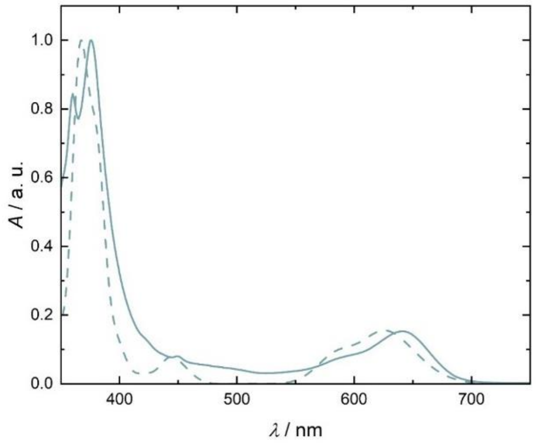

Figure S25: Experimental (solid line) and calculated absorption spectrum (dashed line) of **5,7-ICz-TTM₂**. The calculated spectrum results from the combination of the triplet and open-shell singlet spectra of both (*P*,*M*)-**5,7-ICz-TTM₂** and (*P*,*P*)-**5,7-ICz-TTM₂**.

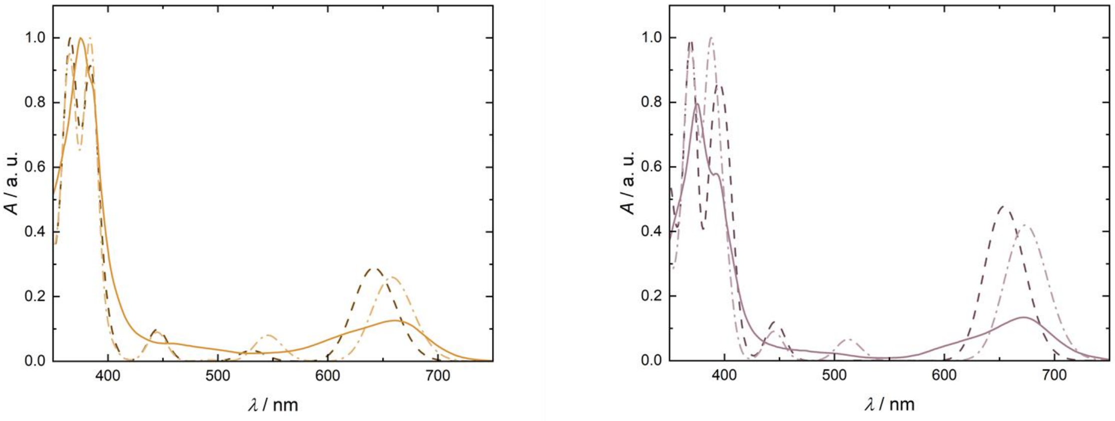

Figure S26: Experimental (solid lines) and calculated absorption spectra of the triplet (dashed lines) and open-shell singlet (dashed dotted lines) state of **5,8-ICz-TTM₂** (left) and **5,11-ICz-TTM₂** (right).

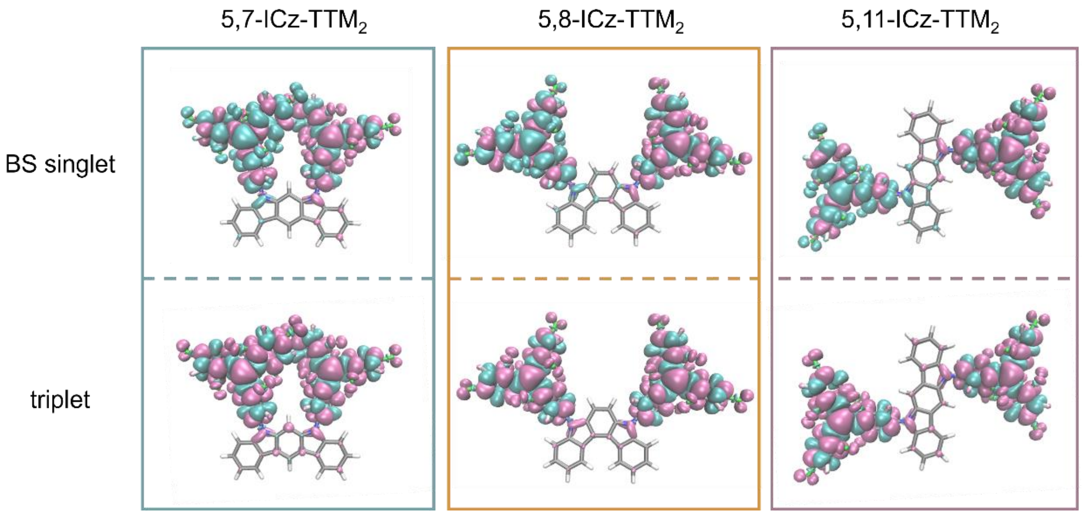

Figure S27: A spin density map for **5,7-**, **5,8-**, and **5,11-ICz-TTM₂** obtained with the PBE0-GD3zero/def2-TZVP level of theory with a CPCM solvation model to simulate a cyclohexane environment. (isovalue = 0.0004).

For further results of the calculations, please look up on Zenodo.org: DOI: 10.5281/zenodo.15488688.

6. EPR Measurements

The EPR spectrum of Br-TTM was measured at 293 K using a Bruker e-scan operating in the X-band (9.83 GHz) with quartz EPR tubes. For this measurement, the modulation frequency was set to 86 kHz and the modulation amplitude to 0.096 mT at a microwave power of 52.7 mW (17 dB). Field correction was performed using solid 2,2-diphenyl-1-picrylhydrazyl (DPPH) as a standard (g = 2.0036). The LANDÉ-factor of Br-TTM results in g = 2.0048.

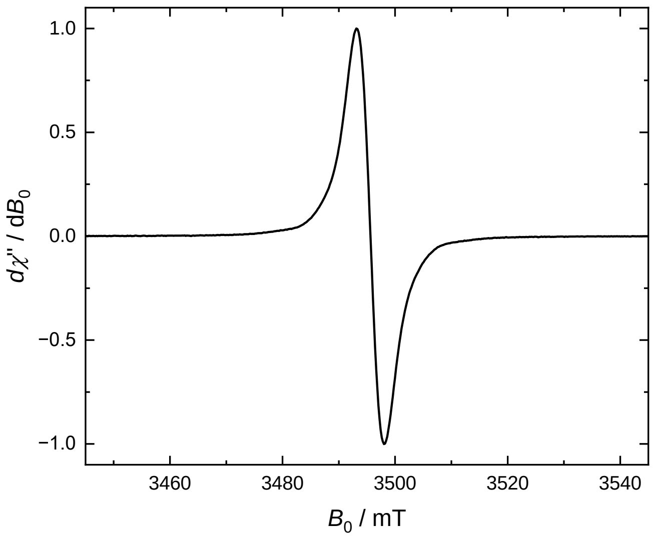

Figure S28: EPR spectrum of **Br-TTM** (solid, 293 K).

6.1 EPR Setup

Electron Paramagnetic Resonance (EPR) measurements of the diradicals were performed at the X-band (9.75 GHz) in quartz EPR tubes with an outer diameter of 3.8 mm (inner diameter of 3 mm), and at the Q-band (34 GHz) in quartz EPR tubes with an outer diameter of 1.6 mm (inner diameter of 1 mm).

Continuous wave (cw) EPR spectroscopy was performed on a Bruker ELEXSYS E580 EPR spectrometer equipped with a Bruker ER4118X-MD5 (X-Band) resonator. Measurements were carried out in liquid toluene solution at room temperature and in frozen toluene solution at 80 K using an Oxford Instruments nitrogen gas-flow cryostat (CF 935). The recorded spectra were frequency-corrected to 9.75 GHz and field-corrected using a carbon fiber standard with g = 2.002644.[19]

Variable-temperature continuous wave (cw) EPR spectroscopy was performed on a Bruker ELEXSYS E500 EPR spectrometer using liquid helium. The sample was stabilized for one hour at 5 K and subsequently for 20 minutes at each temperature. For measurements of the half-field line the modulation frequency was set to 100 kHz and the modulation amplitude to

0.2 mT at a microwave power of 20.24 mW (10 dB). The recorded spectra were background-corrected and frequency-corrected to 9.75 GHz.

Pulse EPR spectroscopy was performed on a Bruker ELEXSYS E580 EPR spectrometer equipped with a Bruker EN45107D2 (Q-Band) or a Bruker ER4118X-MD5 (X-Band) resonator. Measurements were carried out at 80 K using an Oxford Instruments nitrogen gas-flow cryostat (CF 935). Electron spin echo (ESE) detected field sweep spectra were recorded at the Q-Band using the Hahn-echo sequence, $\pi/2 - \tau - \pi - \tau -$ echo, with a 32 ns π-pulse. The field-swept PEANUT measurement was performed at the X-Band using the PEANUT pulse sequence, (π/2, x, 60 ns) – τ – (HTA, x, t) – (HTA, -x, T-t) – τ – echo, [20–23] where the first entry refers to the style of pulse, the second refers to the phase of the pulse and the third refers to the pulse length. The high-turning-angle (HTA) pulse is subdivided into two parts that differ in phase by π. [20–23] A π/2-pulse of 60 ns is used in the measurement, to achieve narrow excitation and therefore fine resolution of the nutation frequencies in field dimension. The recorded data were frequency-corrected to 9.75 GHz and field-corrected using a carbon fiber standard with g = 2.002644.[19]

6.2 EPR Characterization of the Diradicals

The in solution X-Band EPR measurements result in the isotropic LANDÉ-factors g_1 = 2.0034, g_2 = 2.0036 and g_3 = 2.0036 for **5,7-**, **5,8-** and **5,11-ICz-TTM₂**, respectively.

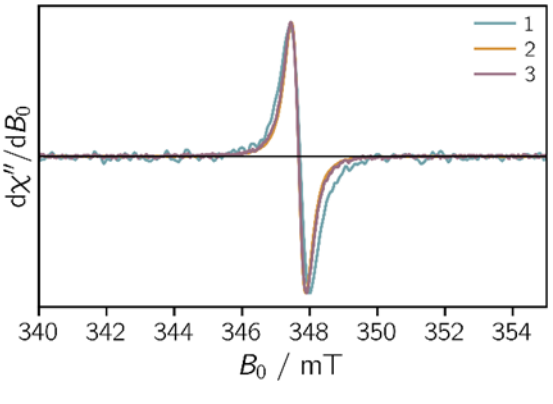

Figure S29: EPR spectra of **5,7-**, **5,8-** and **5,11-ICz-TTM$_2$** in liquid toluene solution at 293 K.

The frozen solution spectra of **5,7-ICz-TTM2** shown in the main part were simulated using EasySpin (v6.0.5) [16] in MATLAB (vR2023b). The powder spectra of the triplet species were simulated using the function 'pepper'. The simulations were globally fitted to the

experimental X- and Q-Band spectra using the 'esfit' function and the NELDER-MEAD simplex algorithm. The resulting simulation parameters are summarized in Table S4.

Table S4: Simulation parameters for the triplet species of **5,7-ICz-TTM$_2$** and the monoradical impurity

	**5,7-ICz-TTM$_2$**	impurity
S	1	½
g	[2.0026, 2.0042, 2.0037]	2.0035
**|*D*| / MHz**	125.2	-
**|*E*| / MHz**	5.5	-
***HStrain_X* / MHz**	[18, 18, 18]	-
***HStrain_Q* / MHz**	[21, 24, 34]	-
***lwpp* / mT**	-	0.5

The EPR spectra of **5,7-**, **5,8-** and **5,11-ICz-TTM$_2$** may also be simulated as a coupled spin system consisting of two spin $S = ½$. For compound **5,7-ICz-TTM$_2$** the value of the exchange coupling constant *J* determined by variable temperature EPR is used in the simulation. *J* could not be determined for the other two compounds **5,8-ICz-TTM$_2$** and **5,11-ICz-TTM$_2$**, using this method, and is assumed to be zero for the realization of the simulation. The spectra are shown in Figure S30 and the simulation parameters are summarized in Table S5.

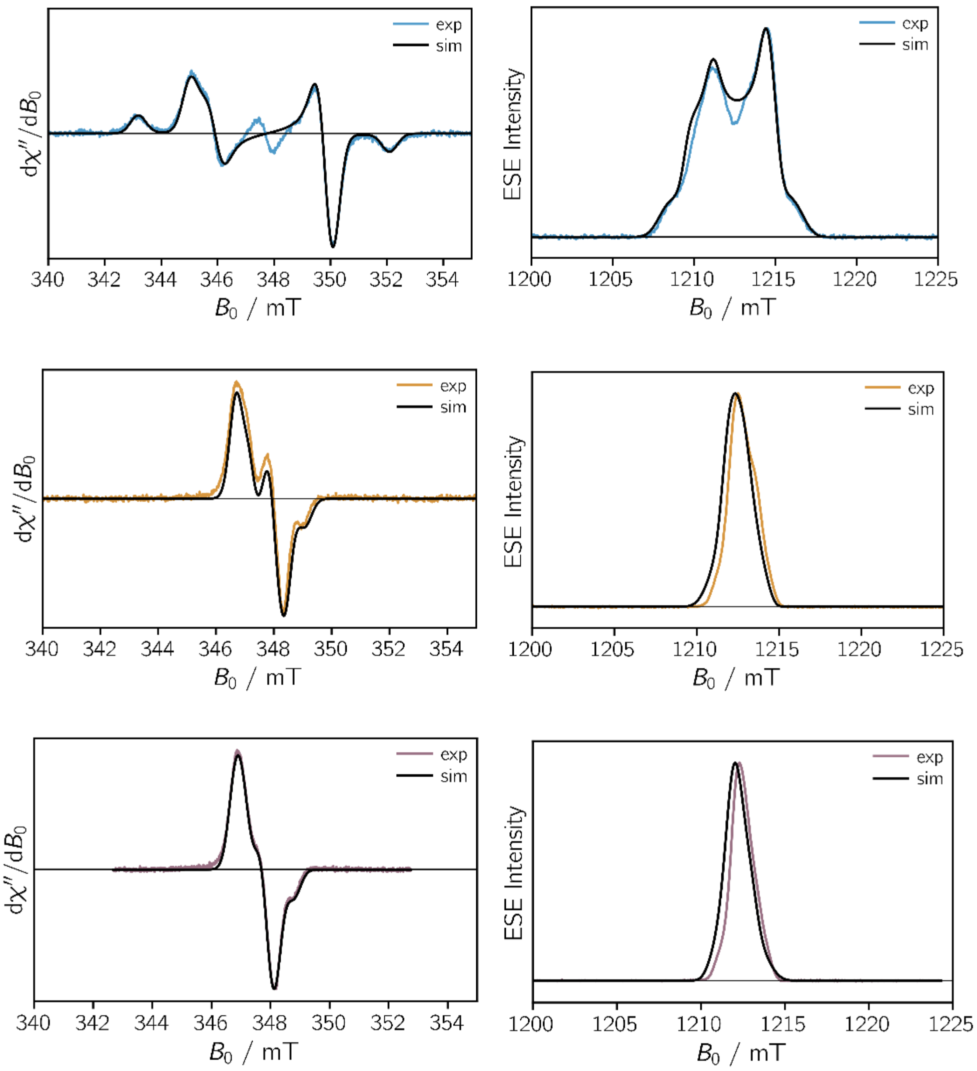

Figure S30: Experimental cw X-band EPR spectra (left) and electron spin echo (ESE) detected Q-band EPR spectra of **5,7-** (top, turquoise), **5,8-** (mid, orange) and **5,11-ICz-TTM₂** (bottom, purple) measured at 80 K in frozen toluene solution with simulations (black) using a coupled spin system for each compound. The simulation parameters of the simulations are summarized in Table S5.

Table S5: Simulation parameters for the coupled spin system simulation of **5,7-**, **5,8-** and **5,11-ICz-TTM₂**

	**5,7-ICz-TTM₂**	**5,8-ICz-TTM₂**	**5,11-ICz-TTM₂**
S	[½; ½]	[½; ½]	[½; ½]
g	[2.0027, 2.0043, 2.0034; 2.0022, 2.0043, 2.0042]	[2.0020, 2.0053, 2.0032; 2.0031, 2.0057, 2.0019]	[2.0038, 2.0053, 2.0036; 2.0028, 2.0062, 2.0019]
***J* / MHz**	130320	0	0
***dip* / MHz**	[83.4, -10.2]	[22.3, -2.2]	[17.5, -1.7]
***HStrain_X* / MHz**	[18, 18, 18]	[13, 13, 16]	[14, 15, 15]
***HStrain_Q* / MHz**	[20, 20, 35]	[20, 35, 20]	[20, 25, 35]

The dipolar interaction is characterized by the axial (dip_{ax}) and rhombic (dip_{rh}) dipolar interaction parameters, which correspond to the first and second entry given in Table S5, respectively. The axial dipolar coupling can be considered the strength of the dipolar interaction and is proportional to the inverse cube of the distance d between the two radical centers, while the LANDÉ-factors are approximately taken to be isotropic ($g_{iso}=(g_x+g_y+g_z)/3$) for each of the coupled spins (g_A, g_B):[24]

$$dip_{\mathrm{ax}} = \frac{\mu_0\,\mu_\mathrm{B}^2\,g_\mathrm{A}\,g_\mathrm{B}}{4\pi\,h}\frac{1}{d^3}$$

where μ_0 is the permeability of the vacuum, μ_B the BOHR magneton and h is PLANCK's constant, which is used here because dip_{ax} is given in frequency units (Hz). Using the simulation parameters in Table S5 allows the determination of the inter-spin distances presented in the main part (Table 1). The following serves as an example calculation for **5,7-ICz-TTM₂**.

$$dip_{\mathrm{ax}} = \frac{\mu_0\,\mu_\mathrm{B}^2\ \cdot 2.0035\ \cdot\ 2.0036}{4\pi\,h}\frac{1}{d^3} = \frac{5.2104\cdot 10^{-20}\ \mathrm{Hz\,m^3}}{d^3}$$

$$d = \sqrt[3]{\frac{52.104\ \mathrm{MHz\,nm^3}}{83.4\ \mathrm{MHz}}}\ =\ 0.85\ \mathrm{nm}$$

6.3 Variable-Temperature EPR evaluation

The temperature-dependent signal intensities of the half-field transition ($\Delta m_s = \pm 2$) were fitted using the relation: [20–23]

$$I\ \propto\ \frac{2g^2\mu_\mathrm{B}{}^2}{3k_\mathrm{B}T}\left[1+\frac{1}{3}\exp\frac{-J}{k_\mathrm{B}T}\right]^{-1}$$

where I is the signal intensity (obtained from numerical double-integration, cf. Figure 4b), g the isotropic LANDÉ-factor of the triplet species, μ_B the BOHR magneton, k_B the BOLTZMANN constant, T the temperature and J the exchange coupling constant. Here the prefactor –1 is used in the definition of the exchange coupling Hamiltonian ($\hat{H}_J = -J\ s_1 s_2$). Using this definition, the singlet-triplet energy gap corresponds to the absolute value of J ($\Delta E_{\mathrm{ST}} = |\,J\,|$), positive values of J correspond to ferromagnetic coupling ($E_\mathrm{S} > E_\mathrm{T}$) and negative values correspond to anti-ferromagnetic coupling ($E_\mathrm{S} < E_\mathrm{T}$). Note that varying definitions of $\hat{H}_J$ are used throughout the literature which differ in the prefactor to J. Careful care must be taken when comparing literature values.

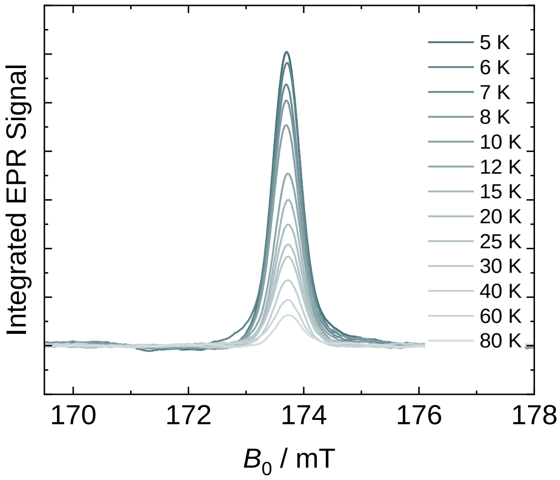

Figure S31: Numerically integrated temperature-dependent X-Band EPR spectra of the half-field transition of compound **5,7-ICz-TTM₂** after background correction. A second numerical integration yields the area value used as a measure for the signal intensity.

6.4 Additional PEANUT data

The field-swept PEANUT experiment yields oscillatory signals shown in Figure S32. Fourier transformation yields the frequency-domain dataset (Figure 4c), where the frequency axis was normalized by the reference frequency obtained from the residual signal of a monoradical impurity at 347.7 mT.

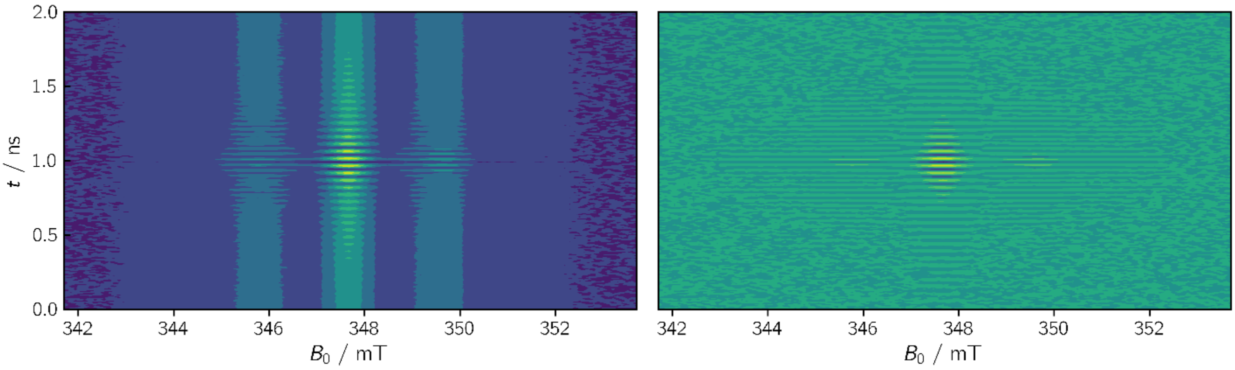

Figure S32: Frequency and field corrected raw data of the field-swept PEANUT experiment of compound **5,7-ICz-TTM$_2$**, before (left) and after (right) background correction.

7. Mass and NMR Spectra

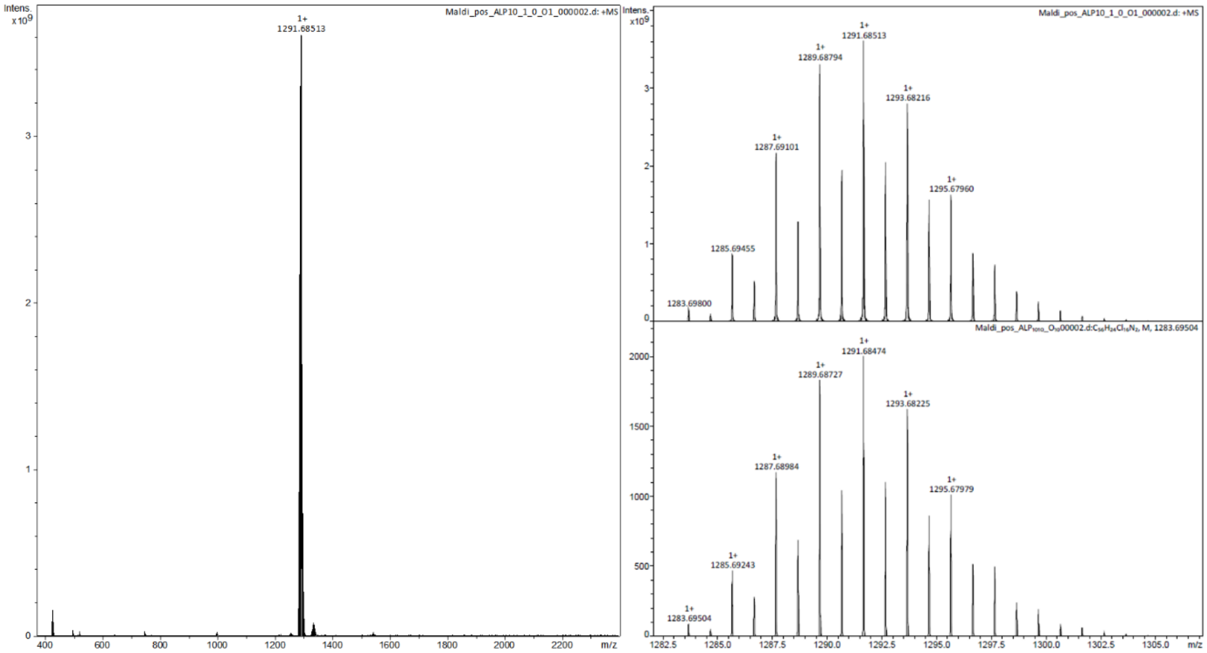

Figure S33: Left: HRMS MALDI FTICR spectrum of **5,7-ICz-HTTM₂** (Matrix: DCTB). Upper right: Experimental spectrum in the range of *m z*$^{-1}$ = 1282.5 – 1305.5. Lower right: Calculated spectrum in the range of *m z*$^{-1}$ = 1282.5 – 1305.5.

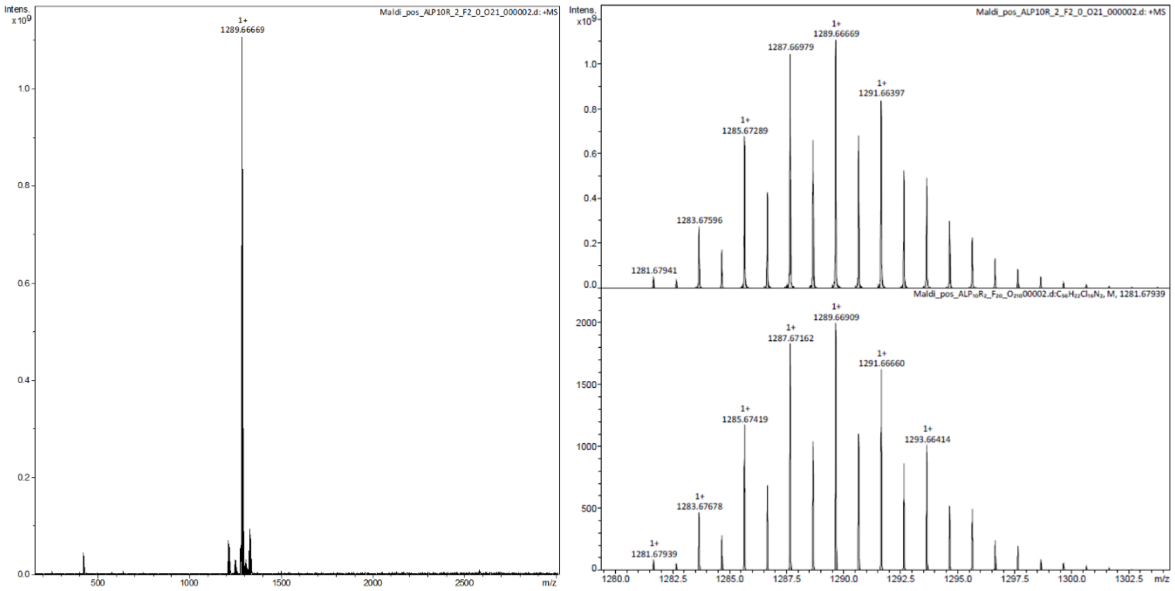

Figure S34: Left: HRMS MALDI FTICR spectrum of **5,7-ICz-TTM₂** (Matrix: DCTB). Upper right: Experimental spectrum in the range of *m z*$^{-1}$ = 1282.0 – 1302.5. Lower right: Calculated spectrum in the range of *m z*$^{-1}$ = 1282.0 – 1302.5.

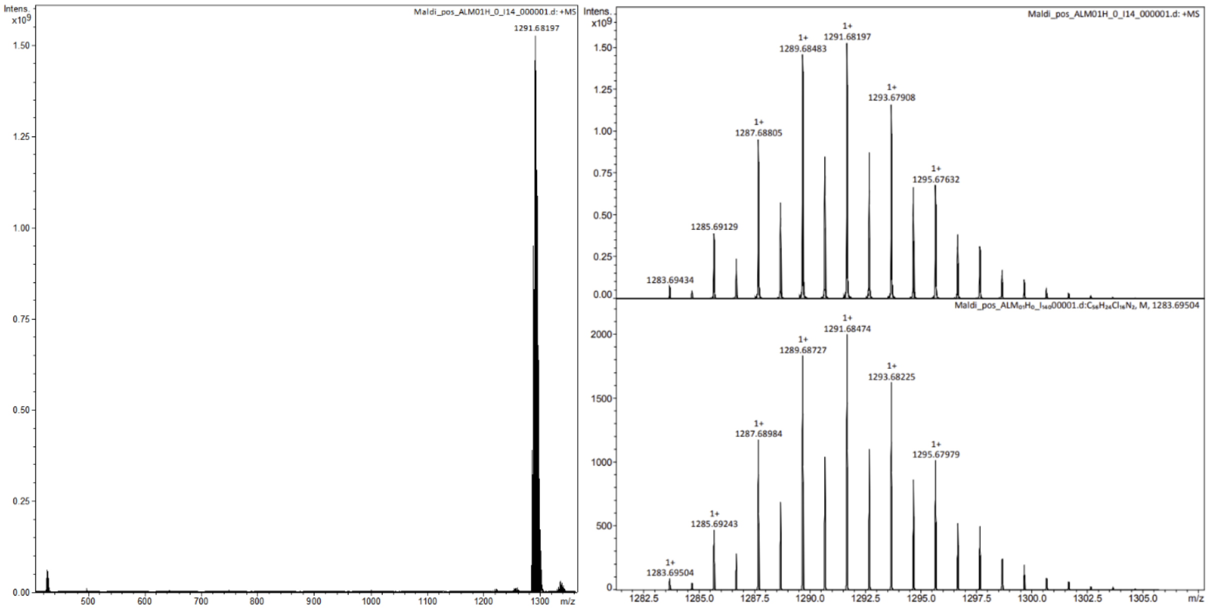

Figure S35: Left: MALDI FTICR spectrum of **5,8-ICz-HTTM₂** (Matrix: DCTB). Upper right: Experimental spectrum in the range of $m\ z^{-1}$ = 1280.0 – 1302.5. Lower right: Calculated spectrum in the range of $m\ z^{-1}$ = 1282.5 – 1302.5.

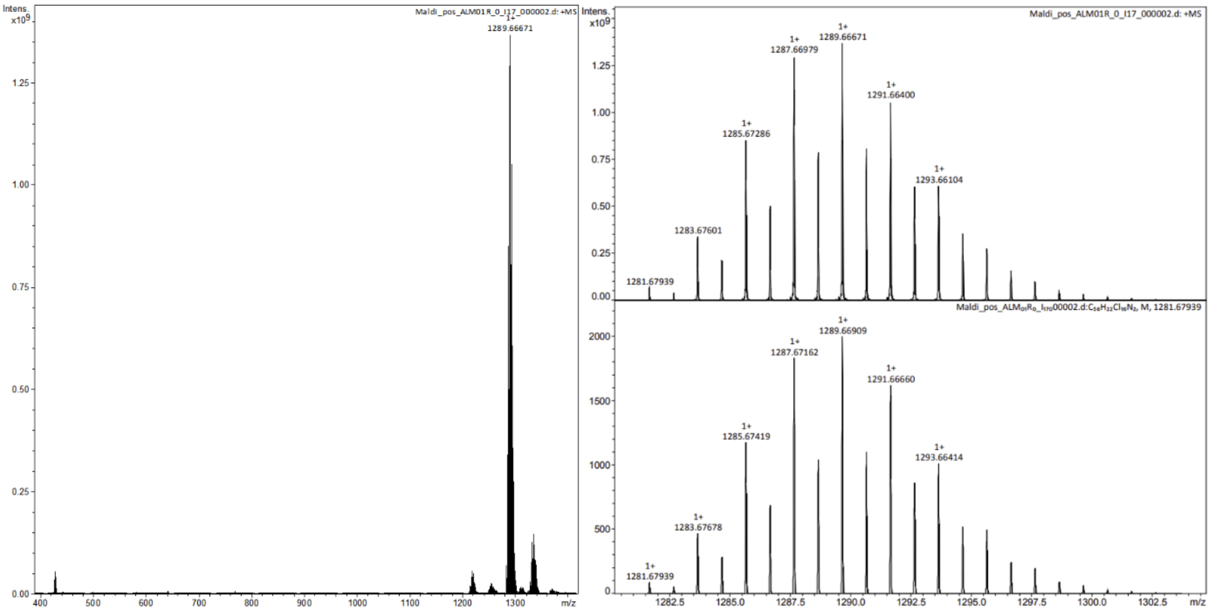

Figure S36: Left: MALDI FTICR spectrum of **5,8-ICz-TTM₂** (Matrix: DCTB). Upper right: Experimental spectrum in the range of $m\ z^{-1}$ = 1282.0 – 1302.5. Lower right: Calculated spectrum in the range of $m\ z^{-1}$ = 1282.0 – 1300.5.

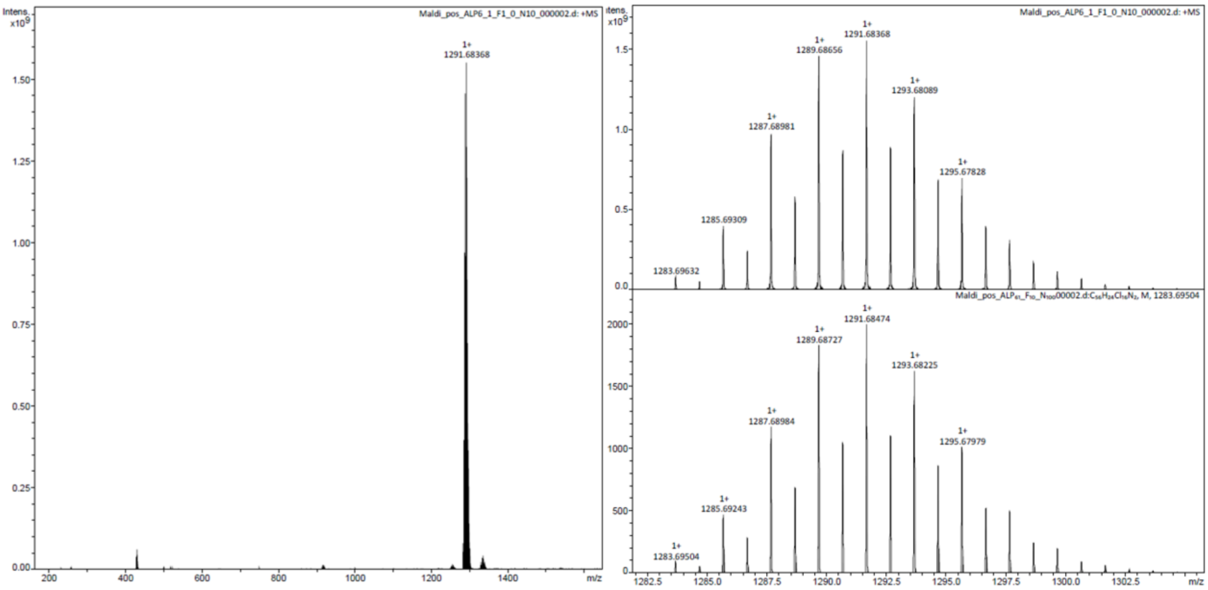

Figure S37: Left: HRMS MALDI FTICR spectrum of **5,11-ICz-HTTM₂** (Matrix: DCTB). Upper right: Actual spectrum in the range of *m* z^{-1} = 1282.5 – 1302.5. Lower right: Calculated spectrum in the range of *m* z^{-1} = 1282.5 – 1302.5.

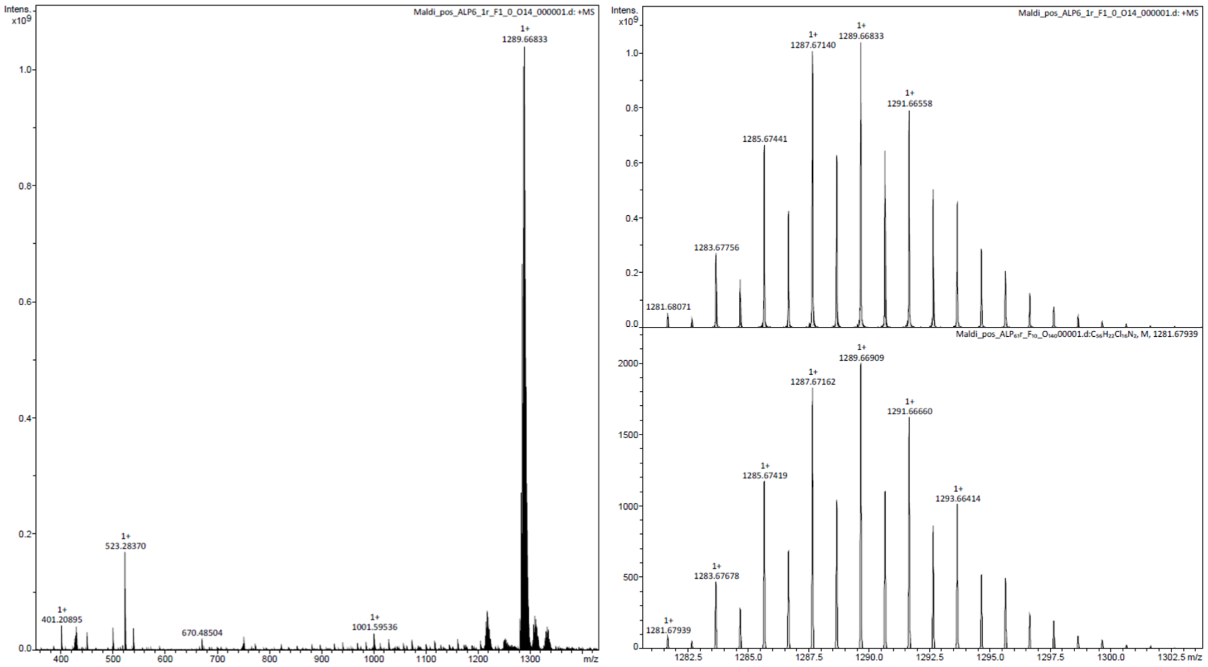

Figure S38: Left: HRMS MALDI FTICR spectrum of **5,11-ICz-TTM₂** (Matrix: DCTB). Upper right: Experimental spectrum in the range of *m* z^{-1} = 1282.5 – 1302.5. Lower right: Calculated spectrum in the range of *m* z^{-1} = 1282.5 – 1302.5.

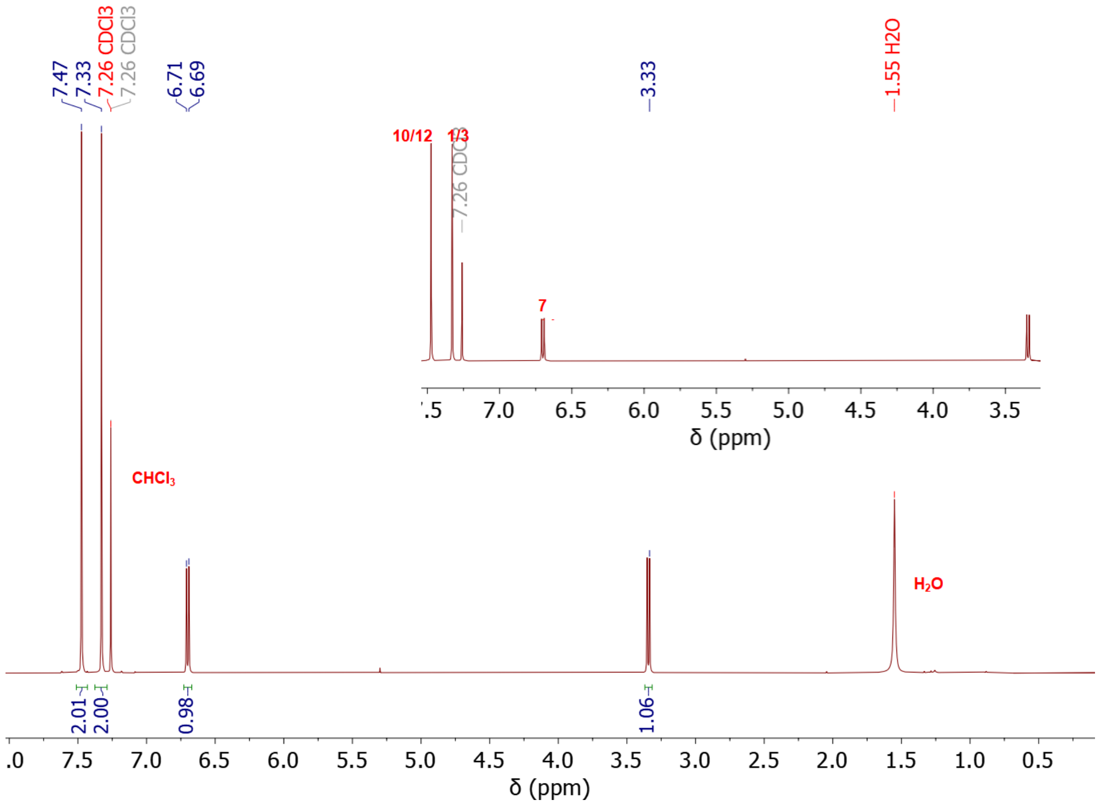

Figure S39: ^{1}H NMR spectrum of **a** (600 MHz, $CDCl_3$, 293 K).

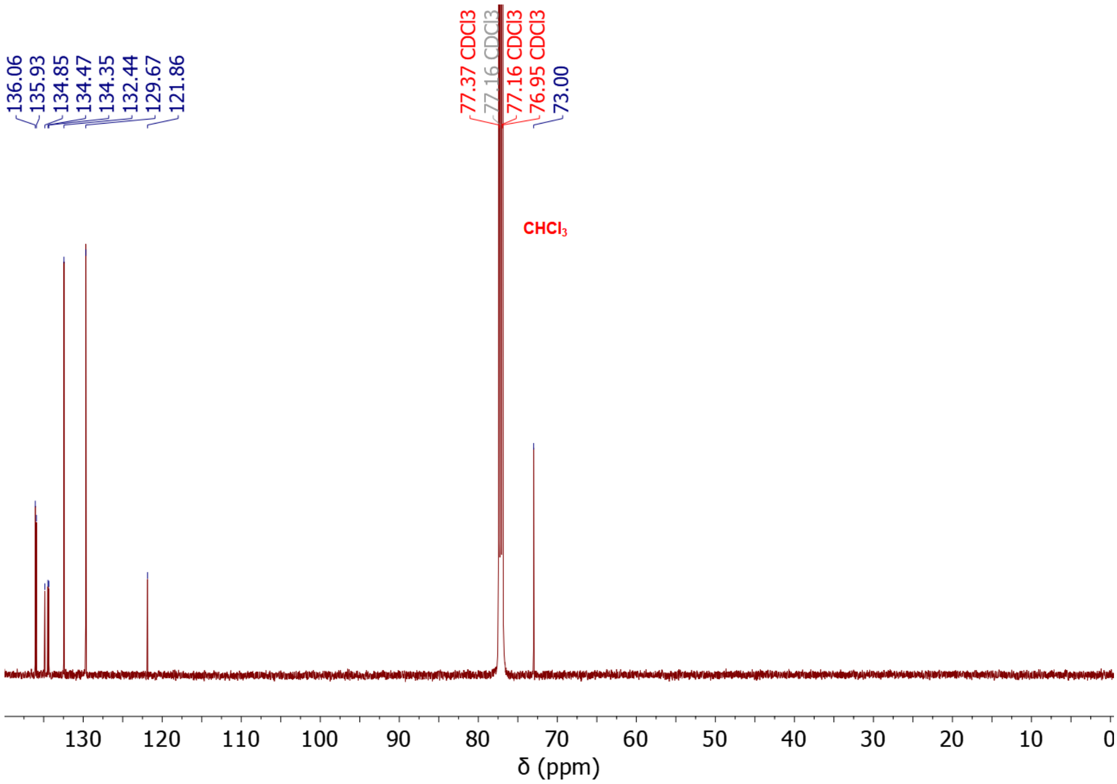

Figure S40: $^{13}C\{^{1}H\}$ NMR spectrum of **a** (151 MHz, $CDCl_3$, 293 K).

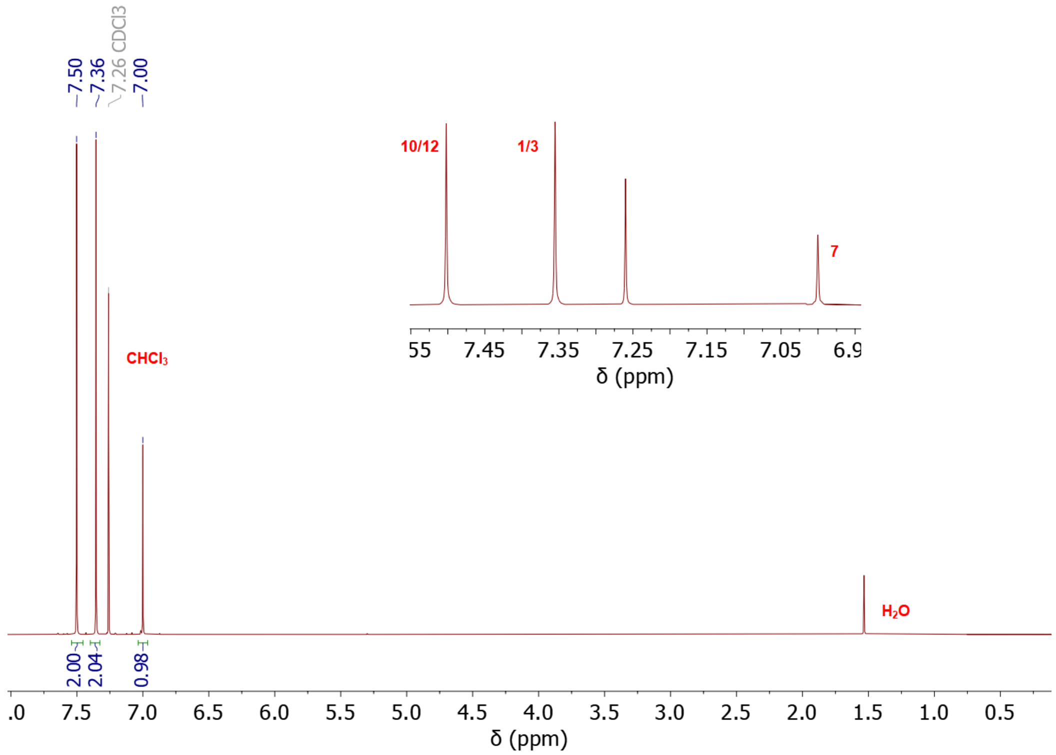

Figure S41: ^{1}H NMR spectrum of **b** (600 MHz, $CDCl_3$, 293 K).

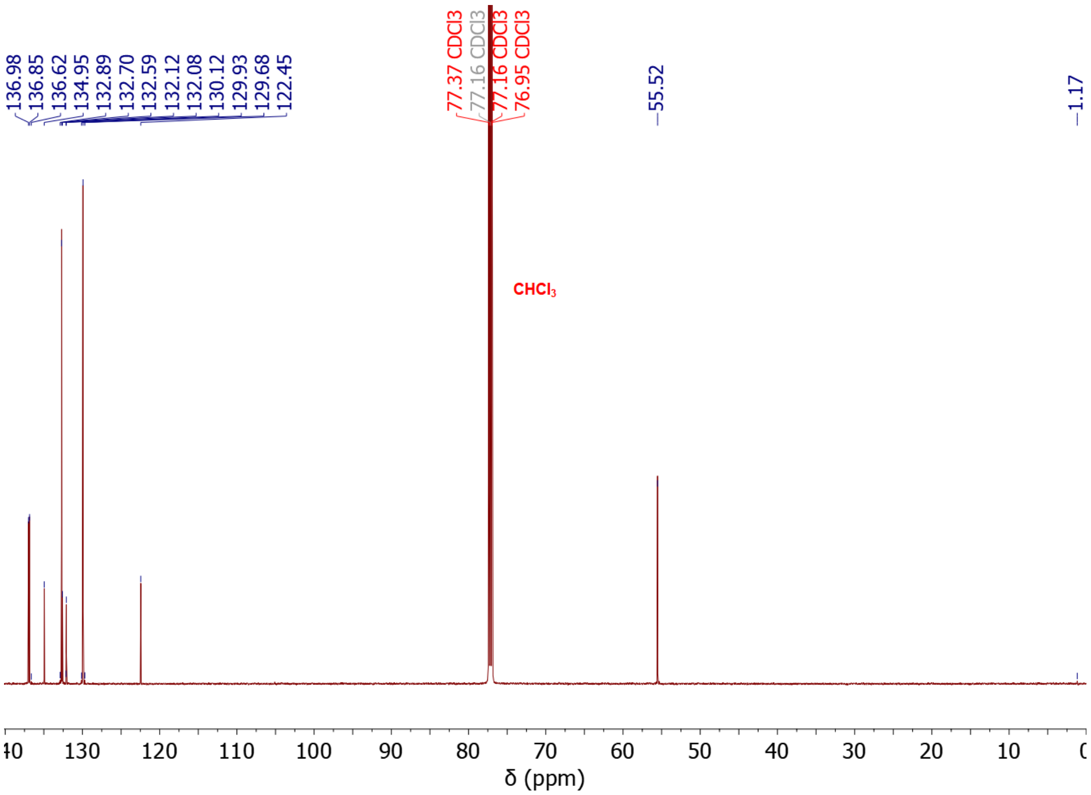

Figure S42: ^{13}C{^{1}H} NMR spectrum of **b** (151 MHz, $CDCl_3$, 293 K).

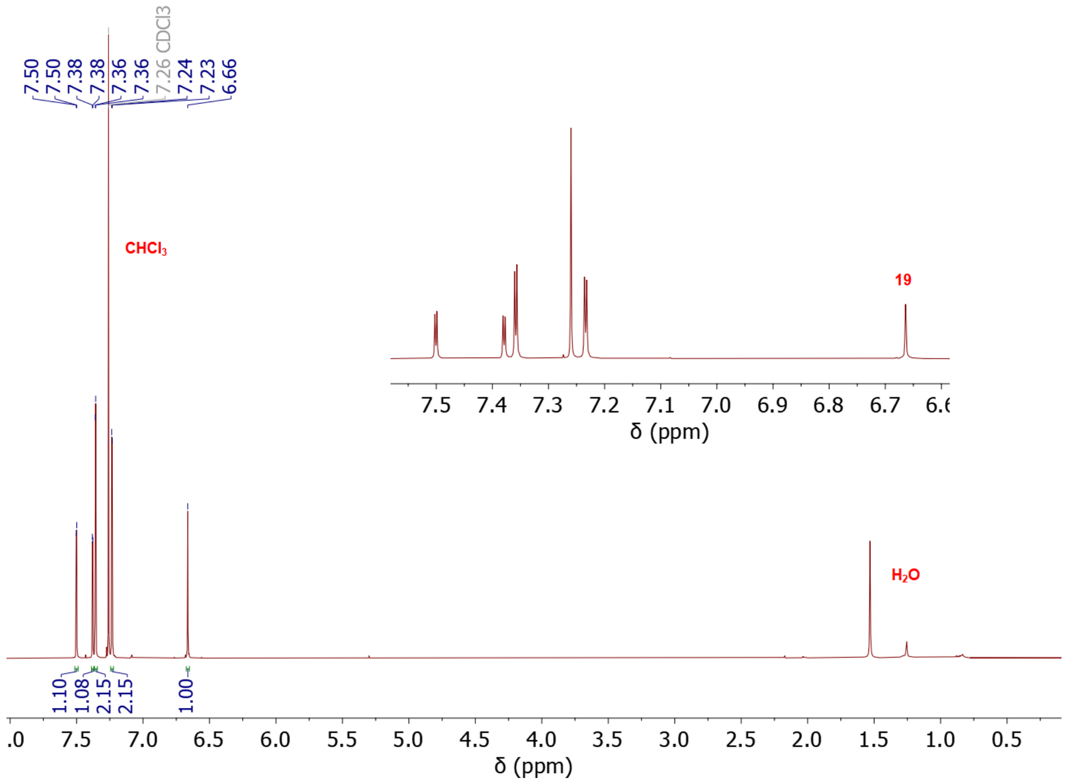

Figure S43: ^{1}H NMR spectrum of **Br-HTTM** (600 MHz, $CDCl_3$, 293 K).

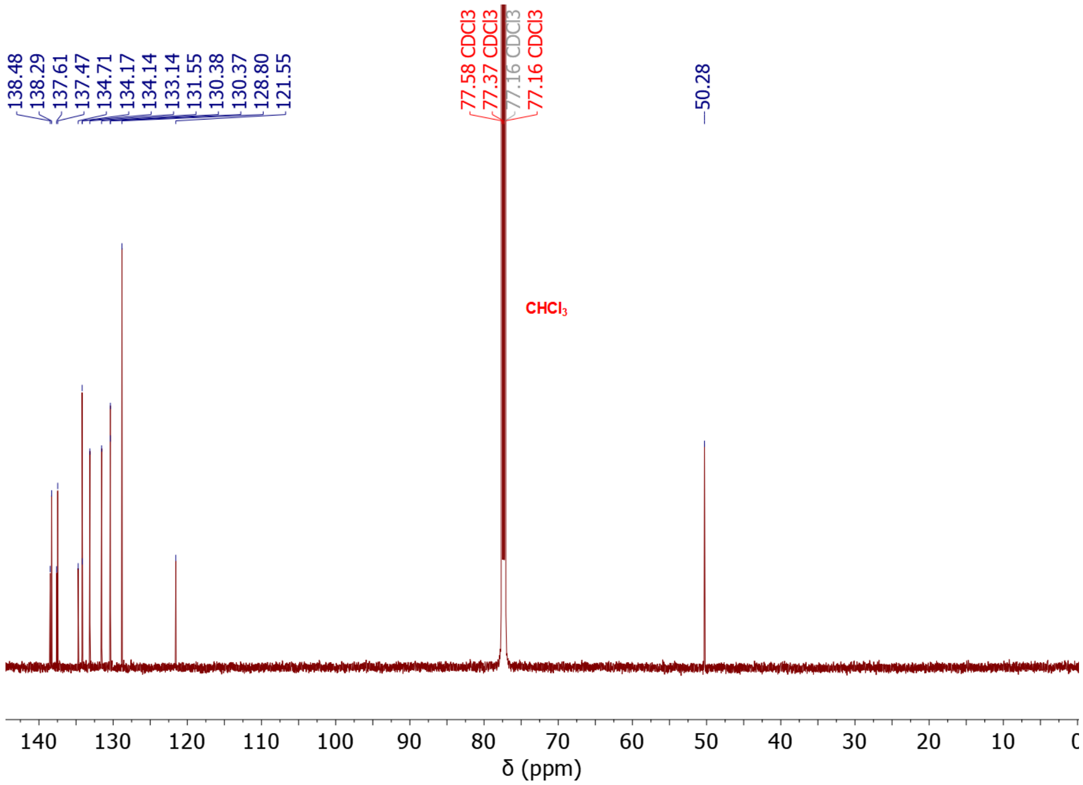

Figure S44: $^{13}C\{^{1}H\}$ NMR spectrum of **Br-HTTM** (151 MHz, $CDCl_3$, 293 K).

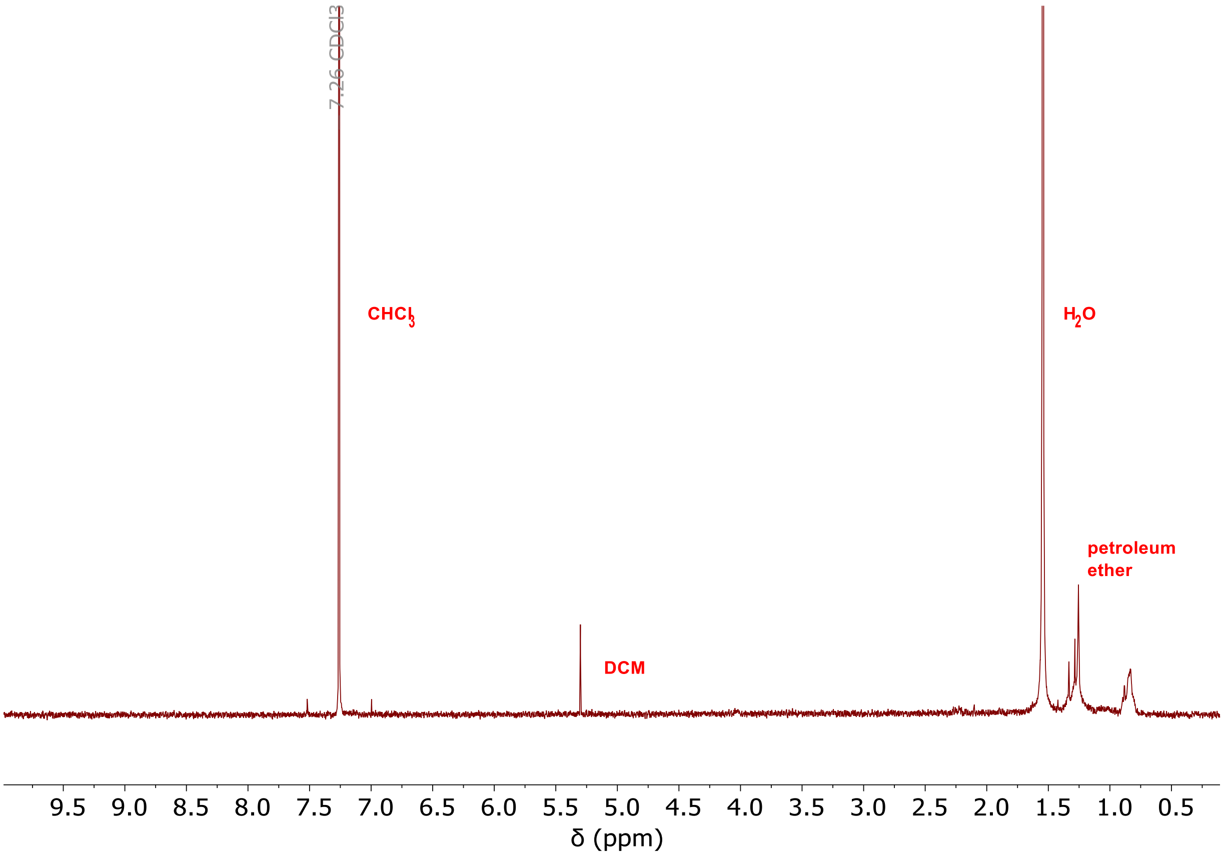

Figure S45: ^{1}H NMR spectrum of ~5 mg **Br-TTM** (400 MHz, $CDCl_3$, 293 K).

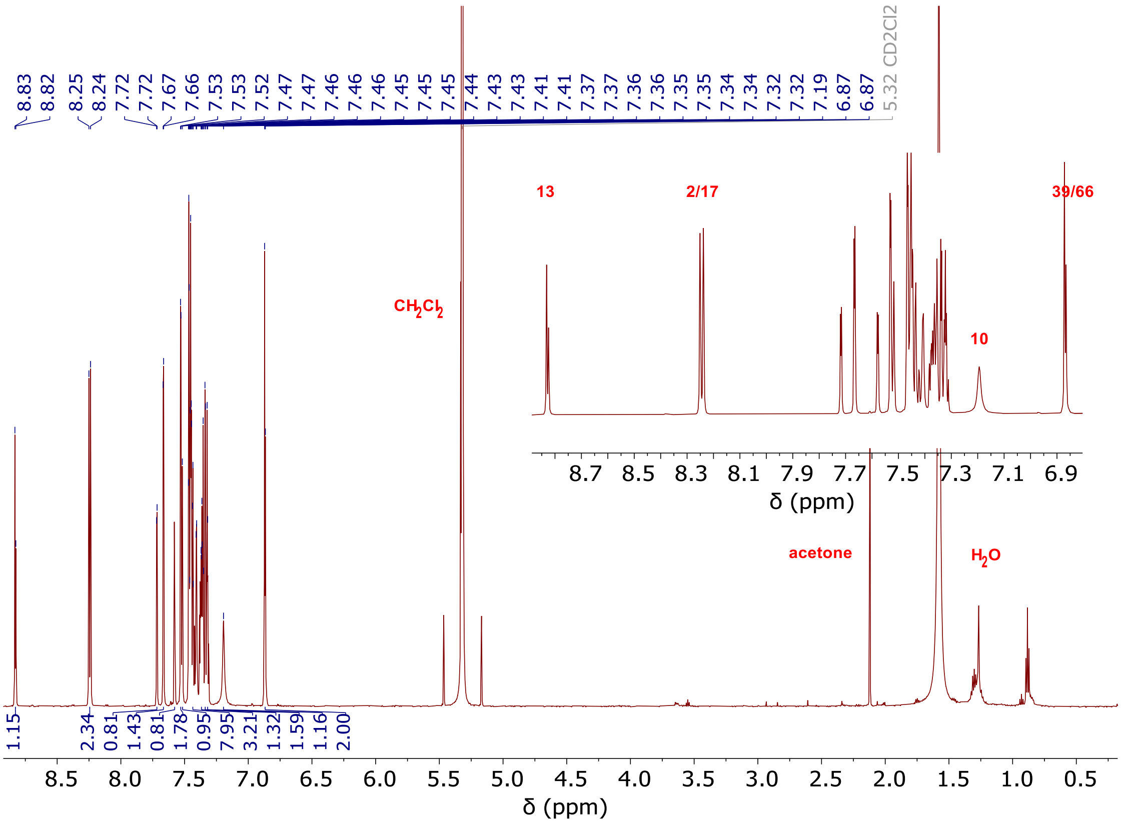

Figure S46: ^{1}H NMR spectrum of **5,7-ICz-HTTM₂** (600 MHz, CD_2Cl_2, 293 K).

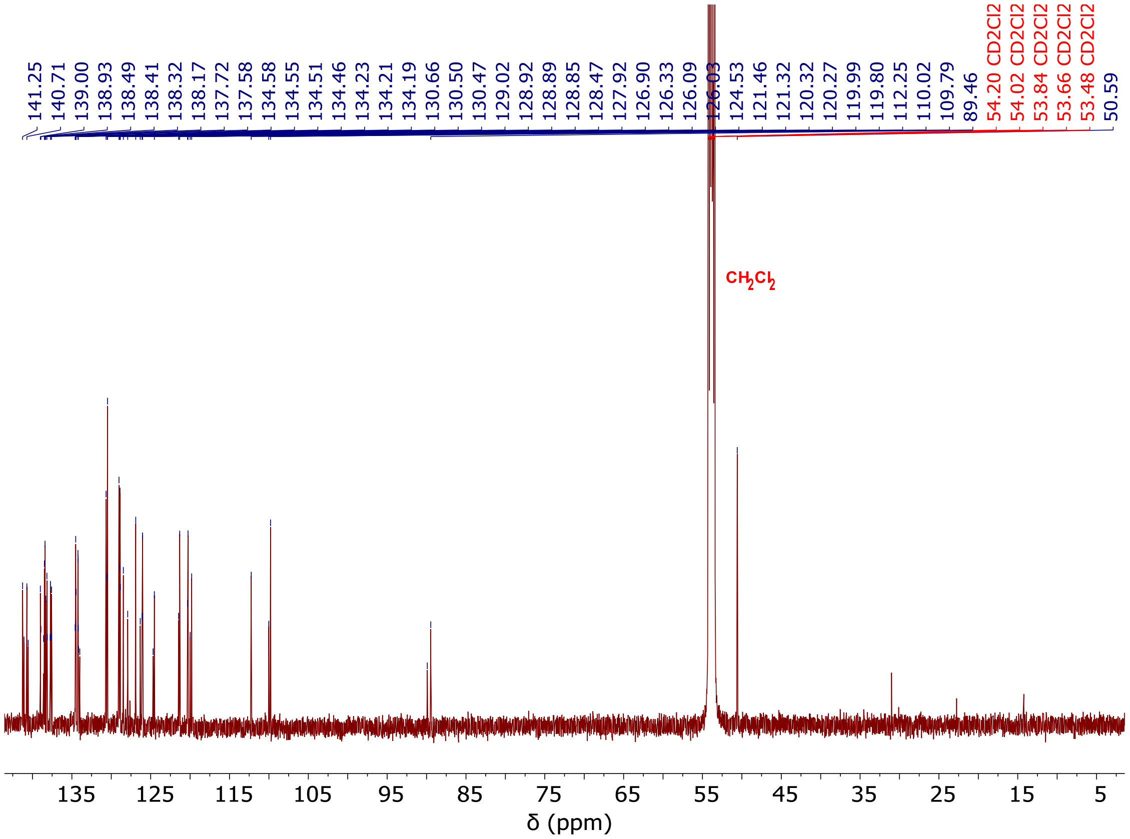

Figure S47: $^{13}C\{^{1}H\}$ NMR spectrum of **5,7-ICz-HTTM₂** (151 MHz, CD_2Cl_2, 293 K).

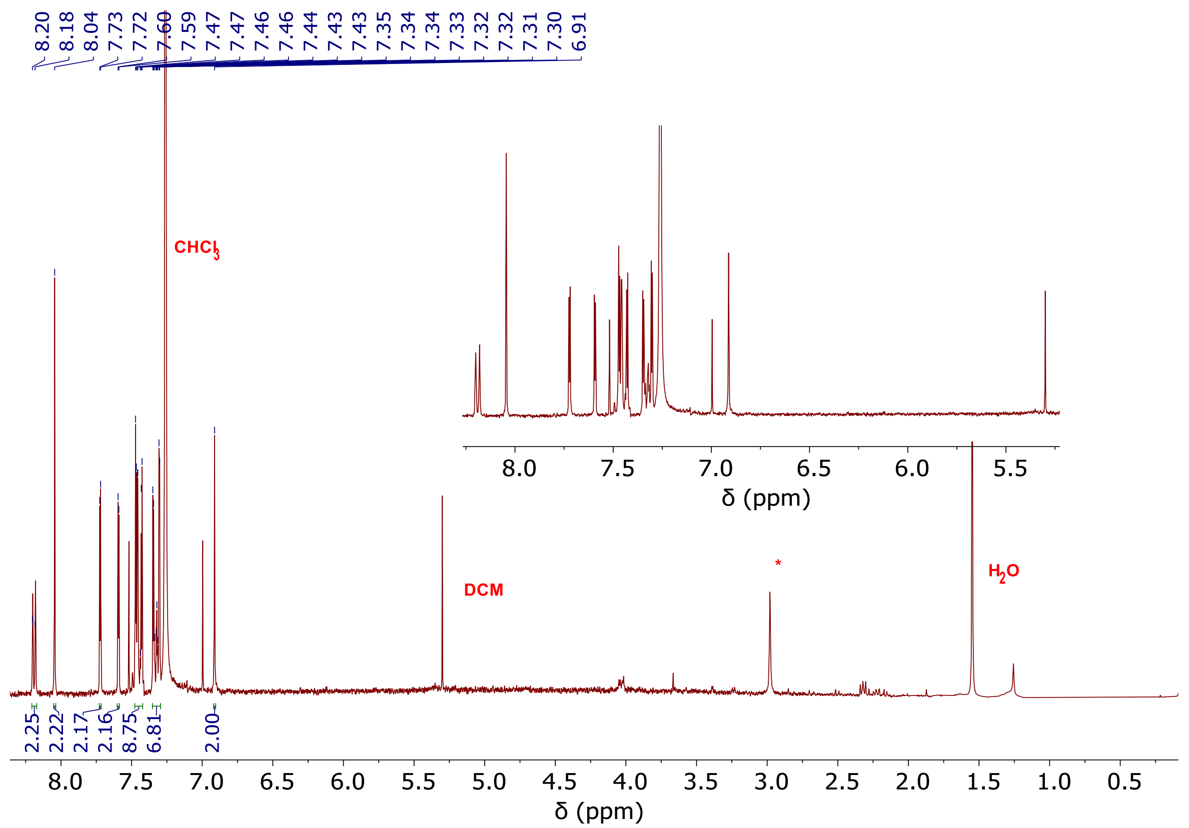

Figure S48: ^{1}H NMR spectrum of **5,11-ICz-HTTM₂** (600 MHz, $CDCl_3$, 293 K). Solvent impurities are labelled with *.

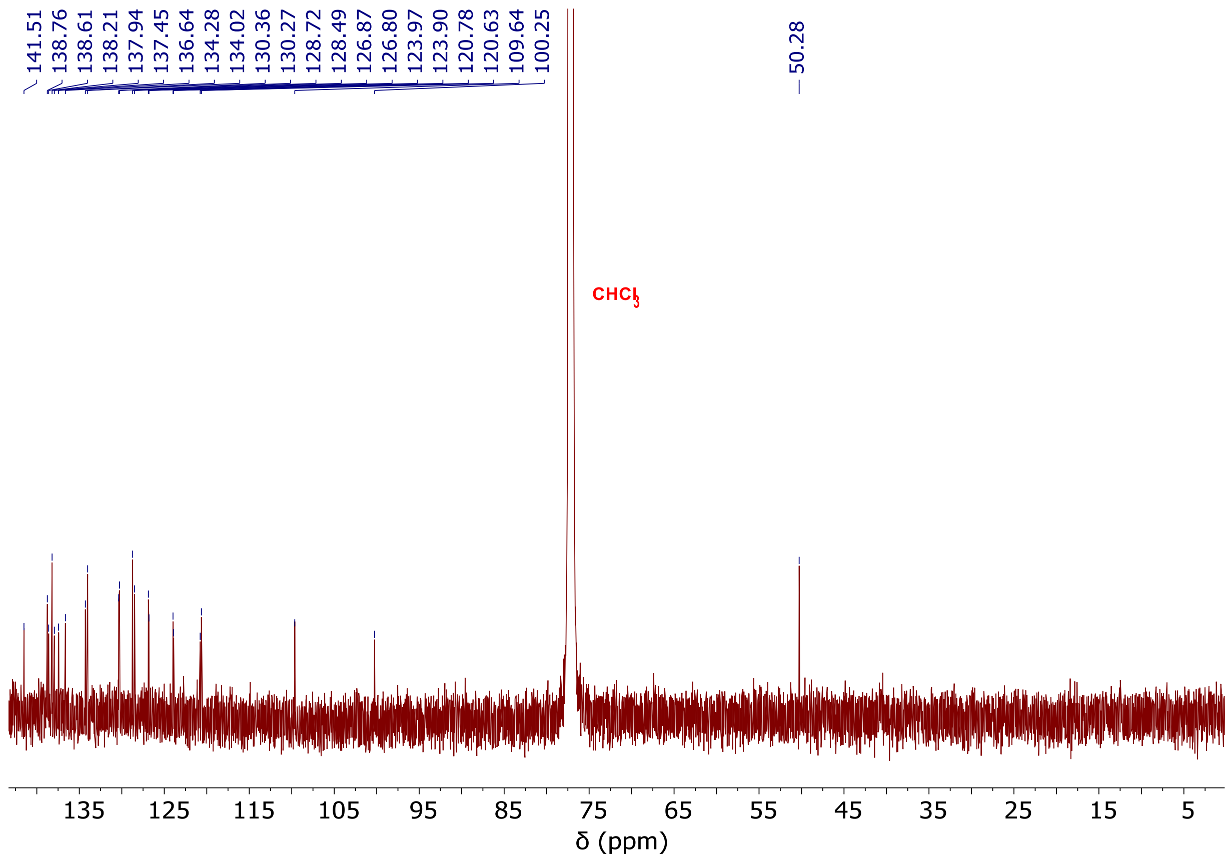

Figure S49: $^{13}C\{^{1}H\}$ NMR spectrum of **5,11-ICz-HTTM₂** (151 MHz, $CDCl_3$, 293 K).

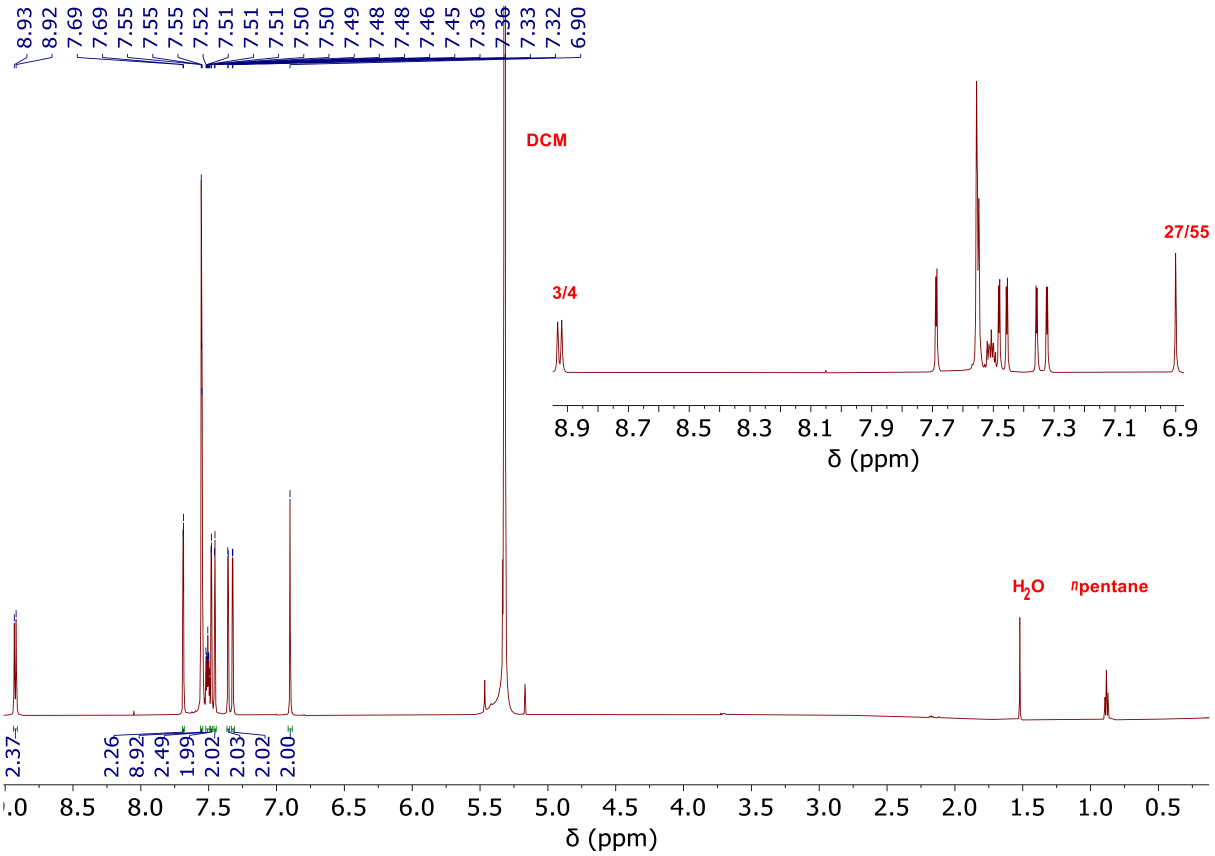

Figure S50: ^{1}H NMR spectrum of **5,8-ICz-HTTM₂** (400 MHz, CD_2Cl_2, 293 K).

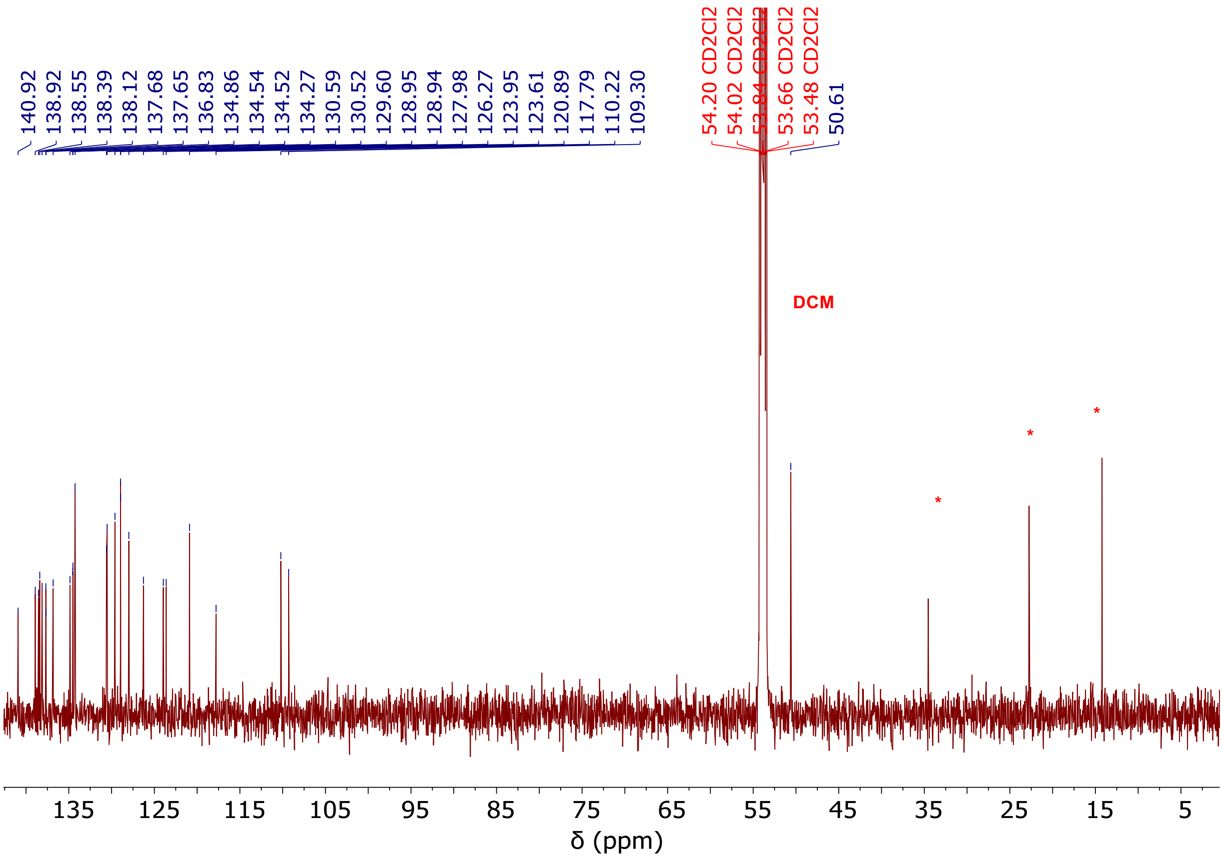

Figure S51: $^{13}C\{^{1}H\}$ NMR spectrum of **5,8-ICz-HTTM₂** (100 MHz, CD_2Cl_2, 293 K). Solvent impurities are labelled with *.

References

[1] M. E. Arnold, A. J. C. Kuehne, *Dyes and Pigments* **2023**, *208*, 110863.

[2] A. Abdurahman, L. Shen, J. Wang, M. Niu, P. Li, Q. Peng, J. Wang, G. Lu, *Light Sci Appl* **2023**, *12*, 272.

[3] V. C. Wakchaure, X. Chang, J. Zolg, R. Blinder, M. E. Arnold, F. Jelezko, A. J. C. Kuehne, M. von Delius, *Chemical Communications* **2025**, DOI 10.1039/D5CC01805F.

[4] S. Gorgon, K. Lv, J. Grüne, B. H. Drummond, W. K. Myers, G. Londi, G. Ricci, D. Valverde, C. Tonnelé, P. Murto, A. S. Romanov, D. Casanova, V. Dyakonov, A. Sperlich, D. Beljonne, Y. Olivier, F. Li, R. H. Friend, E. W. Evans, *Nature* **2023**, *620*, 538–544.

[5] S. M. Kopp, S. Nakamura, B. T. Phelan, Y. R. Poh, S. B. Tyndall, P. J. Brown, Y. Huang, J. Yuen-Zhou, M. D. Krzyaniak, M. R. Wasielewski, *J Am Chem Soc* **2024**, *146*, 27935–27945.

[6] C. P. Yu, R. Chowdhury, Y. Fu, P. Ghosh, W. Zeng, T. B. E. Mustafa, J. Grüne, L. E. Walker, D. G. Congrave, X. W. Chua, P. Murto, A. Rao, H. Sirringhaus, F. Plasser, C. P. Grey, R. H. Friend, H. Bronstein, *Sci Adv* **2024**, *10*, DOI 10.1126/sciadv.ado3476.

[7] F. Neese, F. Wennmohs, U. Becker, C. Riplinger, *J Chem Phys* **2020**, *152*, DOI 10.1063/5.0004608.

[8] F. Neese, *Wiley Interdiscip Rev Comput Mol Sci* **2022**, *12*, DOI 10.1002/wcms.1606.

[9] Y. Zhao, D. G. Truhlar, *Theor Chem Acc* **2008**, *120*, 215–241.

[10] F. Weigend, R. Ahlrichs, *Physical Chemistry Chemical Physics* **2005**, *7*, 3297–3305.

[11] F. Weigend, *Physical Chemistry Chemical Physics* **2006**, *8*, 1057–1065.

[12] S. Grimme, J. Antony, S. Ehrlich, H. Krieg, *J Chem Phys* **2010**, *132*, DOI 10.1063/1.3382344.

[13] V. Barone, M. Cossi, *J Phys Chem A* **1998**, *102*, 1995–2001.

[14] M. Ernzerhof, G. E. Scuseria, *J Chem Phys* **1999**, *110*, 5029–5036.

[15] A. V. Marenich, C. J. Cramer, D. G. Truhlar, *J Phys Chem B* **2009**, *113*, 6378–6396.

[16] M. J. Frisch, **2016**.

[17] G. A. Petersson, A. Bennett, T. G. Tensfeldt, M. A. Al-Laham, W. A. Shirley, J. Mantzaris, *J Chem Phys* **1988**, *89*, 2193–2218.

[18] G. A. Petersson, M. A. Al-Laham, *J Chem Phys* **1991**, *94*, 6081–6090.

[19] K. Herb, R. Tschaggelar, G. Denninger, G. Jeschke, *Journal of Magnetic Resonance* **2018**, *289*, 100–106.

[20] J. A. Weil, J. R. Bolton, *Electron Paramagnetic Resonance*, Wiley-Interscience, **2007**.

[21] B. Figgis, R. Martin, *Journal of the Chemical Society (Resumed)* **1956**, 3837–3846.

[22] Bleaney Brebis, K. D. Bowers, *Proc R Soc Lond A Math Phys Sci* **1952**, *214*, 451–465.

[23] P. F. Richardson, R. W. Kreilick, *Copper Complexes with Free-Radical Ligands*, **n.d.**

[24] X. Chang, M. E. Arnold, R. Blinder, J. Zolg, J. Wischnat, J. van Slageren, F. Jelezko, A. J. C. Kuehne, M. von Delius, *Angewandte Chemie - International Edition* **2024**, *63*, e202404853.
